# Multiple Known Mechanisms and a Possible Role of an Enhanced Immune System in Bt-Resistance in a Field Population of the Bollworm, *Helicoverpa zea*: Differences in Gene Expression with RNAseq

**DOI:** 10.3390/ijms21186528

**Published:** 2020-09-07

**Authors:** Roger D. Lawrie, Robert D. Mitchell III, Jean Marcel Deguenon, Loganathan Ponnusamy, Dominic Reisig, Alejandro Del Pozo-Valdivia, Ryan W. Kurtz, R. Michael Roe

**Affiliations:** 1 Department of Biology/Environmental and Molecular Toxicology Program, 850 Main Campus Dr, North Carolina State University, Raleigh, NC 27695, USA; rdlawrie@ncsu.edu; 2 Department of Entomology and Plant Pathology, Campus Box 7647, 3230 Ligon Street, North Carolina State University, Raleigh, NC 27695, USA; jdeguen@ncsu.edu (J.M.D.); loganathan_ponnusamy@ncsu.edu (L.P.); 3 Knipling-Bushland US Livestock Insects Research Laboratory Genomics Center, 2700 Fredericksburg Road, United States Department of Agriculture-Agricultural Research Service, Kerrville, TX 78028, USA; Mitchell.Robert@epa.gov; 4 Department of Entomology and Plant Pathology, Vernon G. James Research & Extension Center, 207 Research Station Road, Plymouth, NC 27962, USA; ddreisig@ncsu.edu (D.R.); adelpozo@ucanr.edu (A.D.P.-V.); 5 Cotton Incorporated, 6399 Weston Parkway, Cary, NC 27513, USA; RKurtz@cottoninc.com

**Keywords:** *Helicoverpa zea*, bollworm, Bt-resistance, insect immunity, Cry1Ac resistance

## Abstract

Several different agricultural insect pests have developed field resistance to Bt (*Bacillus thuringiensis*) proteins (ex. Cry1Ac, Cry1F, etc.) expressed in crops, including corn and cotton. In the bollworm, *Helicoverpa zea*, resistance levels are increasing; recent reports in 2019 show up to 1000-fold levels of resistance to Cry1Ac, a major insecticidal protein in Bt-crops. A common method to analyze global differences in gene expression is RNA-seq. This technique was used to measure differences in global gene expression between a Bt-susceptible and Bt-resistant strain of the bollworm, where the differences in susceptibility to Cry1Ac insecticidal proteins were 100-fold. We found expected gene expression differences based on our current understanding of the Bt mode of action, including increased expression of proteases (trypsins and serine proteases) and reduced expression of Bt-interacting receptors (aminopeptidases and cadherins) in resistant bollworms. We also found additional expression differences for transcripts that were not previously investigated, i.e., transcripts from three immune pathways-Jak/STAT, Toll, and IMD. Immune pathway receptors (ex. PGRPs) and the IMD pathway demonstrated the highest differences in expression. Our analysis suggested that multiple mechanisms are involved in the development of Bt-resistance, including potentially unrecognized pathways.

## 1. Introduction

The bollworm, *Helicoverpa zea* (Lepidoptera: Noctuidae), is a pest of cotton in the United States (US) [1]. Besides cotton, *H. zea* is an economically important pest of corn (also commonly named the corn earworm and tobacco fruitworm), sorghum, wheat, soybeans, and other crops [2,3]. Integrated Pest Management (IPM) uses several different management techniques to control the bollworm. However, an essential component of cotton IPM has been the use of insecticides [2,4]. Because of the intensive use of foliar, chemical insecticides in cotton for many decades to control insect pests, there has been significant public and industry pressure to develop alternative control methods in order to minimize the environmental impact of cotton production. The outcome was the development of Bt-cotton expressing proteins from bacteria that are toxic to insects; this technology is widely used today for pest control in row crops.

Transgenic crops have been an effective alternative to chemical insecticides in IPM. Transgenic Bt-cotton, as one example, has been used commercially for over two decades (since 1996) to control caterpillars (including *H. zea*, the focus of this paper) [3]. Transcripts coding for insecticidal proteins from the bacteria, *Bacillus thuringiensis* (Bt), Cry1Ac, Cry1F, Cry1Ab, and other Cry proteins, were inserted into cotton plants and other crops [3,5]. More recently, Bt-crops have added the Vip family of proteins [3,5]. The latest generation (2015) of US transgenic cotton produces Cry1Ac, Cry1F, and Vip3A [3]. Major benefits of using these insecticidal proteins is their lack of toxicity to humans and other animals, including beneficial insects [6,7]. Effective pest control in cotton is crucial and of special concern now, because resistance has been detected for the Cry toxins [8].

The mechanism of Bt protein toxicity involves ingestion by the insect, followed by protein solubilization, activation via protease cleavage, binding to low-affinity sites, binding to high-affinity sites, toxin insertion into the midgut leading to pore formation, and death by sepsis from gut bacteria invading the insect hemocoel (the insect’s circulatory system) [5,9]. These processes involve interactions with specific proteins that were found in the insect digestive system, including serine proteases and Bt-binding receptors (cadherins, alkaline phosphatases, and aminopeptidases) required for pore formation [5,9]. Bt-transgenic crops and Bt toxic proteins in general have been effective in the control of a variety of pest species, such as Coleoptera (beetles), Diptera (flies and mosquitoes), and Lepidoptera (moths and butterflies) [5,9], either genetically engineered into plants or by topical application.

A major concern in pest management is the development of insecticide resistance. Resistance now to the Cry proteins in multiple pest species has become widespread. In Puerto Rico, populations of the fall armyworm, *Spodoptera frugiperda*, have become resistant to Cry1F in Bt-modified corn [10], and now resistant insects have been found in North Carolina, USA [11,12]. In South Africa, the maize stalk borer, *Busseoloa fusca,* developed resistance to Cry1Ab [13]. Resistance was found for the western corn rootworm, *Diabrotica v. virgifera*, and resistance was found in the pink bollworm, *Pectinophora gossypiella*, in both India and the US [14,15,16]. The first suggestion that caterpillars had the potential to develop resistance to Bt came from the development of a Bt-resistant, tobacco budworm, *Heliothis virescens*, laboratory strain by Dr. Fred Gould at North Carolina State University (NCSU), which was 32,000-fold resistance to Cry1Ac [17,18]. Multi-protein resistance to Cry1A and Cry2A2 was also developed in the laboratory for the fall armyworm at NCSU [17]. The model organism in the studies reported here, *H. zea*, has now developed resistance in the field to transgenic cotton (Cry1Ac [19,20]) and transgenic sweet corn (Cry1A.105, Cry1Ab, and Cry2Ab2 [21,22]) in the US. Resistance to Cry1Ac and Cry1F proteins expressed in recent strains of Bt-cotton have also been reported [3].

Several different mechanisms for insect Bt resistance have been reported for *Heliothis virescens* and in other caterpillars involving changes in toxin activation in the midgut and toxin binding [23,24]. Changes in midgut cadherin receptors, proteases, GPI-anchored alkaline phosphatases, and glycolipid synthesis were shown [23,24,25]. Functional changes in the ATP-binding cassette (ABC) transport proteins was reported [26,27], and a dominant point mutation in tetraspanin proteins (TSPAN) in field resistant *Helicoverpa armigera* was found in China [28]. Decreases is Cry1Ac midgut protease cleavage was described in resistant *H. zea* [29]. A shotgun, global transcriptomics approach was used in this study in order to assess differences in gene expression between field-obtained, Bt-resistant versus laboratory Bt-susceptible (unfed) neonates of *H. zea* both reared under the same laboratory conditions.

## 2. Results

### 2.1. Cry1Ac Susceptibility Bioassays

Cry1Ac feeding bioassays were conducted in order to establish the susceptibility of our field collected Wake Forest (resistant) versus Benzon (susceptible) strains prior to RNA seq. The resistant strain had an LC_50_ of 43.79 µg/cm^2^ while the susceptible strain had an LC_50_ of 0.43 µg/cm^2^ for Cry1Ac [30]. This was a resistance ratio of 100-fold. Statistical analysis (SAS probit analysis) and pertinent data were, as follows, for both bioassays: susceptible (slope = 2.1, 95% CL = 0.26–0.46, Chi-square = 59.44), and resistant (slope = 2.04, 95% CL = 19.36–85.47, Chi-square = 11.01). Confidence limits do not overlap, and therefore the difference in LC_50_ between strains was considered statistically significant.

### 2.2. Genome-Wide Differential Expression in Helicoverpa zea

**Figure 1:**
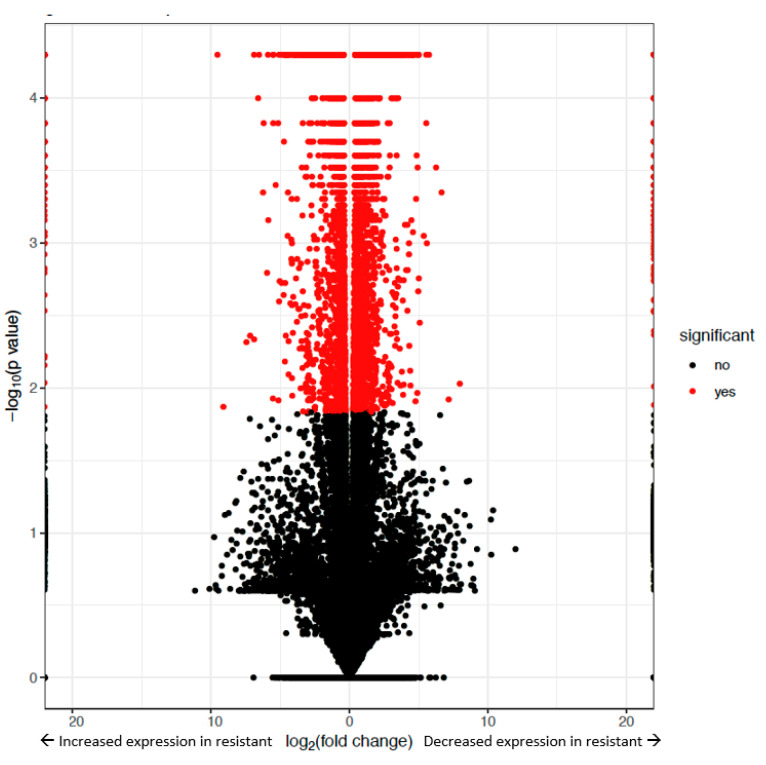
Volcano plot depicting significantly different and insignificantly different transcripts levels determined by RNAseq isolated from *Helicoverpa zea* Bt-resistant and susceptible strains. The *Y*-axis is a −log_10_ (*p* value) scale. The *X*-axis is a log2 (fold change) scale. Each data point indicates a transcript that was differentially expressed between the two strains of caterpillar. Data points to the left of 0 indicate transcripts with increased expression in the resistant strain. Data points to the right of 0 indicate transcripts with decreased expression in the resistant strain. Those in red had statistically significant differential expression levels (α = 0.05). Those in black did not have statistically significant levels of differential expression in these data.

After RNA-Seq, statistically differential expression levels were determined between the resistant versus susceptible strains at α = 0.05. All of the statistically significant differences were included in our analysis, so as not to exclude any possible mechanisms of resistance that might occur when excluding data based on an arbitrary established, minimum fold change. Overall, there were 3056 transcripts with increased expression (up-regulated) and 3042 with decreased expression (down-regulated) in the resistant strain (Figure 1). Additionally, there were 323 expressed transcripts that were found in only the resistant strain and 267 only in susceptible bollworms. The resistant strain had both a higher number of differentially expressed transcripts as well as novel expressed transcripts.

**Figure 2:**
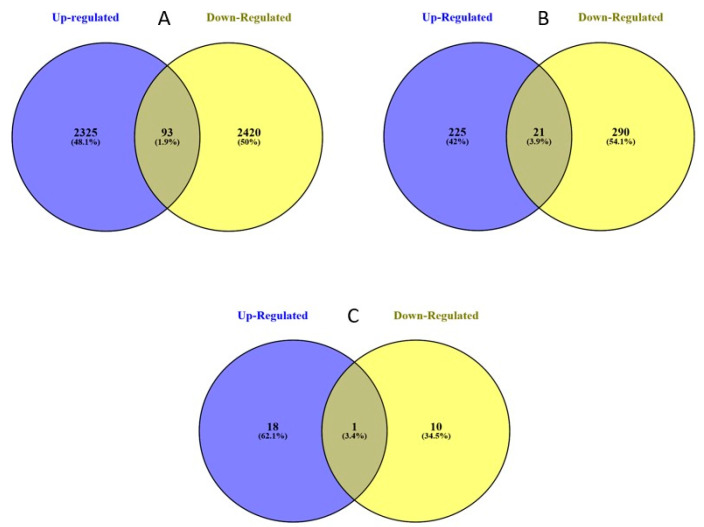
Numbers of statistically significant (α = 0.05) differentially expressed transcripts using 3 different thresholds of log2 fold change for transcripts isolated from *Helicoverpa zea* Bt-resistant and susceptible strains. (**A**) All differentially expressed transcripts, (**B**) those greater than 2.0 log2 fold change in either direction, and (**C**) those greater than 5.0 log2 fold change in either direction. Blue indicates those transcripts with increased expression in the resistant strain. Yellow indicates those transcripts with decreased expression in the resistant strain. Numbers and percentages on the inside of each circle represent the total number of transcripts found to be only up- or down-regulated. Those numbers in the shared section of the Venn diagrams represent the numbers and percentages of transcripts that had copies that were both up- and down-regulated in the resistant strain. These were transcript variants (different mRNA sequences that code for the same protein). Additionally, shared transcripts could have been gene isoforms (mRNA sequences coding for the same protein with differing transcriptional start sites, untranslated regions, or protein coding regions). In some cases, a variant or isoform of a transcript was up-regulated, and in other cases was down-regulated.

We also examined the differential expression between the two strains at different log2 fold change (Figure 2A–C). Those transcripts with a (+) log2 fold change were up-regulated in the resistant strain and those with a (−) log2 fold change were down-regulated. Those transcripts that were unique to each strain were not included in this analysis. Of the differentially expressed transcripts at the α = 0.05 threshold, 2325 (48.1%) were up-regulated, 2420 (50%) down-regulated, and 93 (1.9%) shared (defined in Figure 2 caption) in the resistant strain (Figure 2A). Examining differential expression with a log2 fold change of ≥2.0, 225 (42%) were up-regulated in the resistant strain, 290 (54.1%) down-regulated, and 21 (3.9%) shared (Figure 2B). Examining differentially expression with a log2 fold change of ≥5.0, 18 (62.1%) were up-regulated, 10 (34.5%) down-regulated, and 1 (3.4%) shared in the resistant strain (Figure 2C). The up versus down regulated transcripts were about the same, except when the log2 fold change was ≥5.0, when there was more up-regulation in the resistant strain (Figure 2A–C).

Table 1 shows the top 50 transcripts with the highest degree of up-regulation (log2 fold change) in the resistant strain. Uncharacterized transcripts or those transcripts with no significant matches after a NCBI BLAST search were not included in the top 50 (Figure S1). The highest top 50 log2 fold changes ranged from +9.08 (highest level of up-regulation) to +3.51 log2 fold change (Table 1). General functions of these highest upregulated transcripts were variable. These included, but were not limited to, bromodomain, pupation, serine endopeptidase, metabolic (a broad variety of processes), replication, and binding proteins (Table 1). These top 50 transcripts also contained messages that were previously suggested to be involved in Bt resistance including tetraspanin 1 (+4.9 log2 fold change), serine protease (+5.13), trypsin 3A1 (+5.3), gamma-secretase (+4.7), chymotrypsin 1 (+4.4), and other trypsins (+4.12, +3.94) (Table 1). In general, a number of transcripts implicated before in Bt-resistance were in the top 50 upregulated transcripts; however, some resistance-associated transcripts were not in the top 50 (Table 1).

Table 2 shows the top 50 transcripts with the highest degree of down-regulation (log2 fold change) in the resistant strain (Table 2). Again, uncharacterized transcripts with no matches to *H. zea* after a BLAST search were not included in the top 50 (Figure S2). The highest top 50 log2 fold changes ranged from −7.18 (highest degree of down-regulation) to −3.48 log2 fold change (Table 2). General functions of the top 50 down regulated transcripts include, but were not limited to, xenobiotic metabolism, glucose metabolism, transcriptional modification, binding proteins, transporter proteins, and pupal proteins (Table 2). Present in these top 50 are transcript copies of genes that are known to be involved in resistance. including beta secretase 1 (−3.6 log2 fold change), cytochrome P450s (−7.18, −5.56, −4.54, −4.32, −4.19, −3.86), and alkaline phosphatase (−3.77) (Table 2). Overall, the highest degree of log2 fold change was in the up-regulated category (+9.08) when compared to the down-regulated (−7.18) (Table 1 and Table 2).

### 2.3. Global Gene Functional Annotations for Differentially Expressed Transcripts

**Figure 3:**
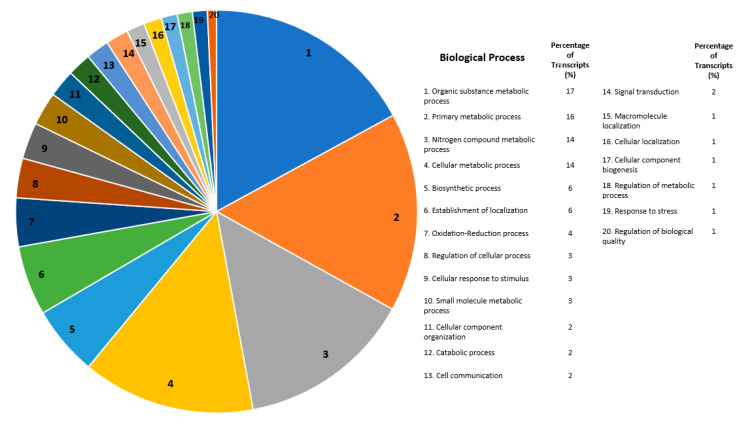
Biological processes gene ontology for all differentially expressed transcripts isolated from *Helicoverpa zea* Bt-resistant versus susceptible strains. Chart on the left depicts the proportion of each biological process. The table to the right is the functional assignments and percentages for each biological process that were differentially expressed. Transcripts were annotated using the OmicsBox program (BLAST2GO function) and were categorized under “Biological process” [31]. Numbers for each biological process on the right (e.g., 1. Organic substance metabolic process) is shown by the respective number on the pie chart to the left.

**Figure 4:**
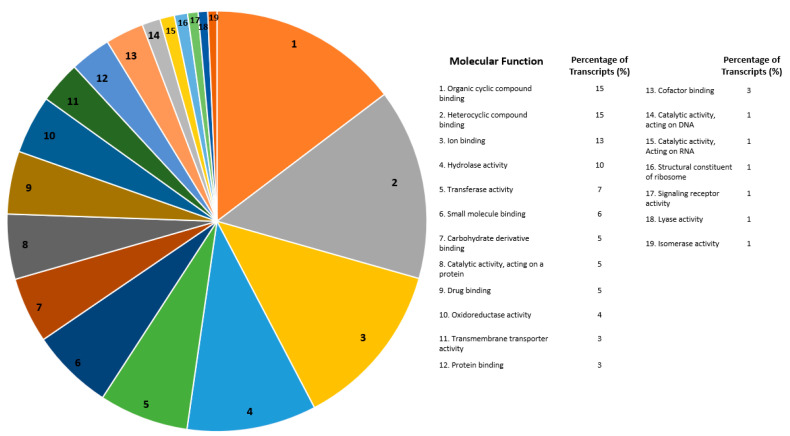
Molecular function gene ontology for all differentially expressed transcripts isolated from *Helicoverpa zea* Bt-resistant versus susceptible strains. The chart on the left depicts the proportion of each molecular function. The table to the right is the functional assignments and percentages for each molecular function that were differentially expressed. Transcripts were annotated using the OmicsBox program (BLAST2GO function) and they were categorized under “Molecular function” [31]. Numbers for each molecular function (e.g., 1. Organic cyclic compound binding) on the right is shown by the respective number on the pie chart to the left.

**Figure 5:**
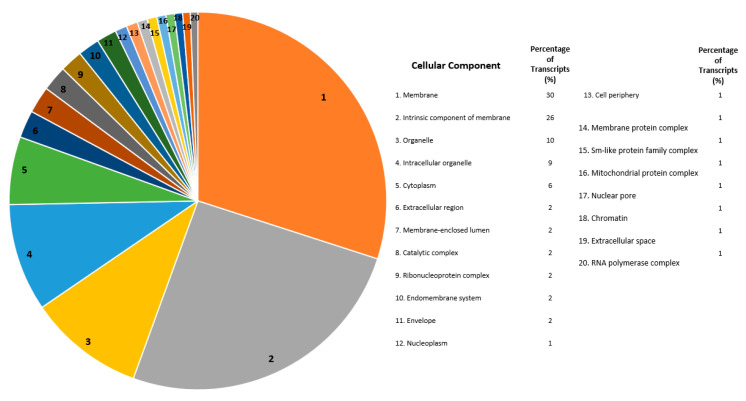
Cellular component gene ontology for all differentially expressed transcripts isolated from *Helicoverpa zea* Bt-resistant versus susceptible strains. The chart on the left depicts the proportion of each cellular component. The table to the right is the functional assignments and percentages for each cellular component that were differentially expressed. Transcripts were annotated using the OmicsBox program (BLAST2GO function) and were categorized under “cellular component” [31]. Numbers for each cellular component (e.g., 1. Membrane) on the right is shown by the respective number on the pie chart to the left.

OmicsBox was used to construct gene ontologies in order to examine functional annotations for all transcripts differentially expressed (Figure 3, Figure 4 and Figure 5). For all differentially expressed transcripts annotated under Biological Processes, transcripts with the greatest number of annotations were defined, as follows: organic substance metabolic process (17%), primary metabolic process (16%), nitrogen compound metabolic process (14%), cellular metabolic process (14%), and biosynthetic process (6%) (Figure 3). Metabolic processes (organic substance, primary metabolism, nitrogen compound, and cellular) were the most prevalent types of processes that were annotated and differentially expressed (Figure 3).

For all differentially expressed transcripts that were annotated under Molecular Functions, transcripts with the greatest number of annotations were defined as follows: organic cyclic compound binding (15%), heterocyclic compound binding (15%), ion binding (13%), hydrolase activity (10%), and transferase activity (7%) (Figure 4). The most prevalent types were binding activity (organic cyclic compound, heterocyclic compound, and ions) and enzymatic activity (hydrolase and transferase) (Figure 4).

For all differentially expressed transcripts annotated under Cellular Components, transcripts with the greatest numbers of annotations were defined, as follows: membrane (30%), intrinsic component of membrane (26%), organelles (10%), intracellular organelle (9%), and cytoplasm (6%) (Figure 5). Under this category, the most prevalent categorizations were transcripts involved in membrane, an intrinsic component of the membrane, or portions of an organelle (Figure 5).

### 2.4. Bt-Resistance Associated Differential Expression

**Figure 6:**
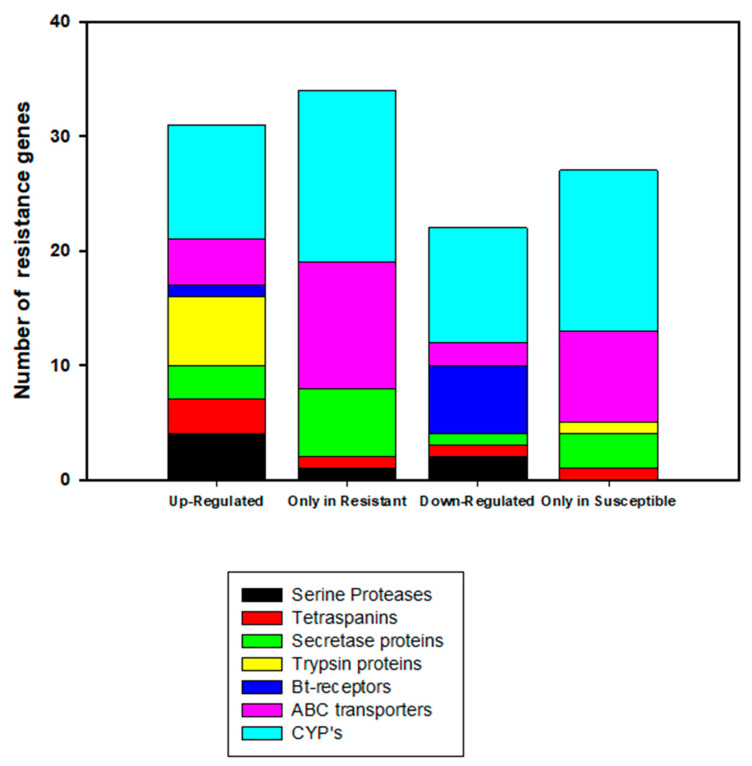
Number of Bt-resistance associated transcripts isolated from *Helicoverpa zea* Bt-resistant and susceptible strains organized by functional category. The Y-axis indicates the total number of transcripts for each category. The X-axis shows the transcripts that were up-regulated or down-regulated in the resistant strain, those found only in the resistant strain, and those found only in the susceptible strain. For those gene families “only in resistant” or “only in susceptible”, these categories represent transcripts that were only present in either of these strains, and therefore were not differentially expressed. Gene families depicted in this chart are as follows: serine proteases (Black), tetraspanins (Red), secretase proteins (Green), trypsin proteins (Yellow), Bt-receptors (Blue), ABC transporters (Purple), and cytochrome P450s (CYP) (Turquoise).

Transcripts that were previously associated with Bt-resistance in lepidopterans were examined for their presence in our bollworm RNAseq results. Figure 6 shows the numbers of transcripts for each category of resistance associated transcripts and their direction of log2 fold change. Table 3 shows specific log2 fold changes for these resistance-associated transcripts with a log2 fold change ≥2.0. The numbers of transcripts and the highest log2 fold change transcripts in these categories are as follows: serine proteases (four up-regulated (+5.13, +6.86, +3.1, and +2.4 log2 fold change), two down-regulated, one only in resistant), tetraspanins (three up-regulated (+4.92, +3.37, and +2.88 log2 fold change), one down-regulated, one only in resistant, one only in susceptible]), secretase proteins (three up-regulated (+4.7, +3.04, and +2.07 log2 fold change), one down-regulated, six only in resistant, three only in susceptible), trypsin proteins (six up-regulated (+4.44, +5.31, +4.12, +3.94, +3.62, and +2.17 log2 fold change), one only in susceptible), Bt-receptors (one up-regulated (+2.93 log2 fold change), six down-regulated (−3.77, −6.26, −2.29, −2.89, −2.02, and −2.21 log2 fold change)), ABC transporters (four up-regulated (+3.36, +3.06, +2.6, and +2.5 log2 fold change), two down-regulated, 11 only in resistant, eight only in susceptible), and cytochrome P450s (CYPs) (10 up-regulated (+4.23, +4.1, +4, +3.37, +2.43, +2.31, +2.22, +2.16, +2.04, and +2.01 log2 fold change), 10 down-regulated, 15 only in resistant, 14 only in susceptible) (Figure 6, Table 3). CYPs were included in this analysis due to evidence showing involvement of these proteins in cross-resistance in insects [32,33,34]. For all the gene families, there were increased expression levels in our resistant bollworm strain with the exception of intestinal receptors (aminopeptidases, alkaline phosphatases, and cadherins), which had decreased expression in resistant bollworms. Beyond these broader protein families, there were also other important potential resistance associated transcripts with high levels of log2 fold change, i.e., carboxypeptidases (+3.04 and −2.03 log2 fold change), chitin synthase (+3.11), heat shock protein (+5.02), carboxyl/choline esterase (+6.17), and E3 ubiquitin-protein ligase associated protein (+5.47) (Table 3).

### 2.5. Differential Expression of Immune Function Associated Transcripts

**Figure 7:**
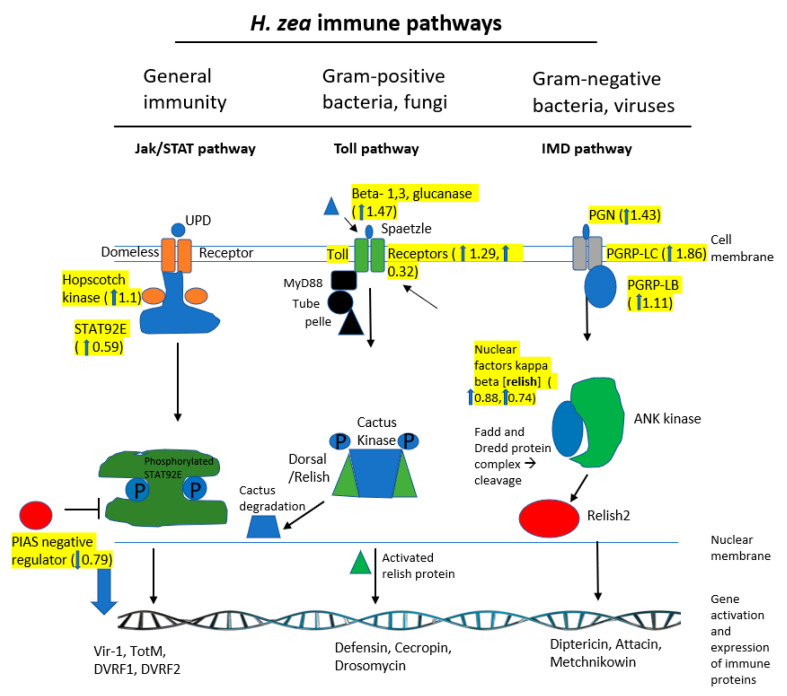
Generalized immune pathways found in *Helicoverpa zea* and differentially expressed transcripts (isolated from Bt-resistant and susceptible strains). Pathway components were found in other research [35,36,37,38]. Three immune pathways are shown, from left to right the Jak/STAT, Toll, and IMD pathways. The function of each pathway is shown at the top of the figure. Direction of the pathways move from top to bottom. The top begins with extracellular receptors or signals, the center corresponds to the cytoplasm, and bottom to the nuclear membrane and DNA level. Shapes indicate a protein involved in one of the pathways and are labelled appropriately. Those proteins highlighted in yellow are transcripts that are involved in any of these immune pathways that were differentially expressed. Magnitude (log2 fold change) and direction (up or down-regulated) of differential expression is indicated in the yellow highlighted section as well.

Figure 7 depicts three generalized immune pathways found in the bollworm: (i) Jak/STAT, (ii) Toll, and (iii) IMD pathways. Table 4 details additional transcripts that are involved in immunity in *H. zea* and their respective log2 fold changes. In the Jak/STAT pathway, three transcripts were differentially expressed: Hopscotch kinase (+1.1 log2 fold increase), STAT92E (+0.59 log2 fold increase), and PIAS (a negative regulator) (−0.79 log2 fold decrease). In the Toll pathway, three transcripts were differentially expressed: beta-1,3, glucanase (+1.47 log2 fold increase) and two Toll receptor proteins (+1.29 and +0.32 log2 fold increase). In the IMD immunity pathway, five transcripts were differentially expressed: PGN (+1.43 log2 fold increase), PGRP-LC (+1.86 log2 fold increase), PGRP-LB (+1.11 log2 fold increase), and two NF-kappa-beta proteins (+0.88 and +0.74 log2 fold increase). In each of these three pathways, the proteins that are involved in cell membrane receptors or protein activation (kinases) were the main types of proteins observed to be differentially expressed. The effector proteins for each pathway (ex. Defensins, attacins) were not found to be differentially expressed (Figure 7). Transcripts involved in the IMD pathway were also observed to have the most differential expression (10 individual transcripts) and also to have the highest degree of increased expression (+3.75 log2 fold change) (Table 4). Beyond the IMD pathway, there were four different transcripts differentially expressed involved in the JAK/STAT pathway, four involved in the Toll pathway, four antimicrobial/bacterial proteins, two pathogen defense or recognition proteins, two immune signaling proteins, six autophagy, and 10 involved in the general immune response (Table 4). Some transcripts involved in immunity also had decreased expression in the resistant strain (ex. Cecropin (−3.39 log2 fold change) and lysozyme (−3.34)); however, overall, the majority of immune-associated transcripts analyzed were found to have increased expression in the resistant strain (Table 4).

## 3. Discussion

### 3.1. Resistant Versus Susceptible Bollworms

There was a 100-fold difference in susceptibility to Cry1Ac between susceptible and resistant bollworms. The optimum comparison would have been the study of both a susceptible and resistant field strain collected from the same location. However, at this juncture, the deployment of GMO crops that express Cry toxins is widespread, Bt resistance is widespread [13,14,15,16], and obtaining a Bt-susceptible field population of bollworms never exposed to Bt selection is not possible. Using a susceptible laboratory strain that is commercially available is advantageous as a standardized reference and it was used before to study new mechanisms of Bt resistance [39,40]. However, some of the differences that we found in this study could be unrelated to Bt resistance but a function of genome selection in a laboratory versus the field. In order to eliminate strain differences as much as possible, both the resistant and susceptible bollworms were reared in the same laboratory at NCSU and on the same artificial diet, under the same environmental conditions, and using the same rearing methods. In addition, we used newly hatched, unfed neonates, so any differences in developmental polymorphisms between the two strains would be minimized. Using unfed neonates also allowed for us to examine the constitutive differences in gene expression between the two strains before stadium develop is initiated by feeding.

### 3.2. Differential Expression Between Bt Resistant Versus Susceptible Bollworms

Initial analysis of the resulting RNA-seq data showed global differences in the gene expression between strains. After removing all non-differentially expressed transcripts and transcript copies, in the resistant strain there were 2325 up-regulated transcripts, 2420 down-regulated, and 93 shared transcripts. Shared transcripts were transcript variants (different mRNA sequences that code for the same protein) or gene isoforms (mRNA sequences coding for the same protein with differing transcriptional start sites, untranslated regions, or protein coding regions). Additional copies or variants of resistance associated transcripts (ex. proteases and transporters) may help to explain how a particular Bt-resistance associated transcript is impacting resistance. In some cases, transcript variants with different mRNA sequences for a gene that code for the same protein were differentially expressed but in opposite directions, for example with cytochrome P450s. Potentially, some transcript variants are impacting Bt-resistance and some are not (discussed in more detail later). When examining the numbers of transcripts with high levels of log2 fold change in resistant neonates, it was found that 225 transcripts were up-regulated, 290 were down-regulated, and 21 were shared using a threshold of ≥2.0 log2 fold change in either direction. Using a threshold of ≥5.0 log2 fold change, 18 transcripts were up-regulated, 10 down-regulated, and one was shared. This indicates that, when comparing each of these different thresholds, only when examining transcripts with the highest degrees of log2 fold change does up-regulation become dominant. An explanation for this could be that the genetic changes that are linked to Bt-resistance are predominantly occurring on specific transcripts instead of global shifts in the genome, which does adhere to the current understanding of Bt-resistance [24,25,28].

The results also indicated a higher overall degree of log2 fold change in the resistant strain (ex. highest log2 fold change +9.08 compared to −7.18 in susceptible neonates). Uncharacterized transcripts with even higher log2 fold change are shown in supplemental Figures S1 and S2. While the functions of transcripts with the highest magnitudes of log2 fold change do not appear to have a known or obvious connection with Bt-resistance (ex. bromodomain protein and cytochrome P450), it may be that the high degree of differential expression is a side-effect of other genetic shifts occurring when Bt-resistance develops in *H. zea* (or a result of strain differences unrelated to Bt resistance). The gene with the highest degree of down-regulation, a cytochrome P450 (CYPs), is normally involved in xenobiotic metabolism and other processes. It would be interesting to examine cross-resistance to insecticide chemistry for Bt resistant bollworms (discussed in more detail later).

### 3.3. Differential Expression of Cytochrome P450s in Bt-Resistant Helicoverpa zea

In examining the CYPs that were differentially expressed, there were 10 up-regulated, 10 down-regulated, 15 only in resistant, and 14 only in susceptible neonates. CYPs are a broad family of proteins that are involved in the metabolism of many different substrates, including endogenous chemicals and xenobiotics [41]. The variation in differential expression for this group of transcripts in this study suggest, in part, that some of these CYPs may have a role in Bt resistance. For example, CYP337Bv1, the gene exhibiting the highest down-regulation may have no role while CYP6B5 with a high degree of up-regulation in the resistant strain (log2 fold change +4.44) may be important in Bt resistance. P450s are not known to metabolize proteins. However, these enzymes are involved in the metabolism of plant secondary compounds and metabolic products from bacteria, among many other substrates [42,43,44]. Perhaps an increase in some P450s is important in Bt resistance, because of their role in the metabolism of secondary metabolites that are produced from the proliferation of bacteria and fungi in the insect hemocoel and/or by the metabolism of secondary plant compounds that reduce insect fitness along with Bt poisoning. However, our study was a comparison between a field vs lab strain; P450 transcript differences could simply reflect differences in xenobiotic exposure and natural selection between living in the field versus being reared on artificial diet. Transcriptional differences in metabolizing enzymes between a laboratory vs a field strain was shown before [45] but cross-resistance between Cry1Ac and the chemical insecticide deltamethrin also was found in the diamondback moth, *Plutella xylostella* L. [46]. Bollworms that were collected from Alachua County, FL (USA) (before the deployment of Bt crops) and reared in the laboratory for three generations had a much higher tolerance to Cry1Ac than other caterpillars collected throughout the SE US (and treated the same) and also demonstrated cross tolerance to Orthene (Roe, personal communication). There were clear differences in CYP transcript levels between Bt resistant and susceptible bollworms in this study, the consequences of which we do not yet understand, but that raise interesting possibilities.

### 3.4. Genome Characterization of Global Differentially Expressed Transcripts

For the differential expression between the resistant and susceptible strains, gene ontologies were constructed to categorize annotated transcripts by function (Figure 3, Figure 4 and Figure 5). Of the differentially expressed transcripts annotated under Biological Processes, the highest proportions were categorized as being involved in organic substance metabolic processes, primary metabolic processes, nitrogen compound metabolism, and cellular metabolic processes. Of the differentially expressed transcripts annotated under Molecular Function, the highest proportions were categorized as being involved in organic cyclic compound binding, heterocyclic compound binding, ion binding, and hydrolase activity. Of the differentially expressed transcripts that were annotated under Cellular Components, the highest proportions were categorized as being involved in the cell membrane, a component of the cell membrane, organelles, and intracellular organelle. These categories include different types of metabolism, binding, and cellular membrane components. Analysis of differential expression levels between Bt-resistant and susceptible *Plutella xylostella* showed similar Gene Ontology results, including metabolic processes, binding, cellular components, and binding processes [46]. A large number of metabolizing transcripts (cytochrome P450s, 49 transcripts) were differentially expressed in our data. Cytochrome P450s are involved in a broad category of metabolic processes and contribute to the high degree of functional categories annotating to metabolic processes. Possible explanations for differential expression in CYPs were discussed in the above section. Further characterization of functional categories for differences in gene expression between resistant and susceptible insects will help to illuminate potential new areas of investigation for resistance mechanisms in the future, especially as more global gene expression studies are conducted related to Bt resistance.

### 3.5. Role of Proteases, Receptors, and Transporters in Resistance

Former studies investigating Bt-resistance in *H. zea* discovered several different mechanisms of resistance. Most importantly, alterations in proteases (secretases, chymotrypsins, and trypsins), midgut Bt-interacting receptors (cadherins, aminopeptidases, and alkaline phosphatases), transporters (ABC), and tetraspanins (TSPAN) have all previously been associated with Bt-resistance [23,24,25,26,27,28]. In this particular study, we found supporting evidence that all of these changes were present in the same resistant strain (increases in proteases, transporters, and tetraspanins; decreases in midgut receptors). Additionally, a number (9) of these important resistance-associated transcripts were found among the top 50 up-regulated transcripts with the highest degree of increased expression in the resistant strain. A mid-gut Bt-interacting receptor (1) was also found to be among the top 50 down-regulated transcripts with decreased expression in the resistant strain. While each of these mechanisms of Bt-resistance have been individually recognized and across several different model organisms, this study represents one of the first to find all of the discussed mechanisms of Bt-resistance present in a single population of *H. zea*. We do not know yet if they all occurred in the same insect. Our results suggest that the resistant population studied evolved Bt-resistance in the field via a wide array of different mechanisms, potentially indicating that the genomic control mechanisms for insecticide resistance may fall under the same control pathway. Ideally, as investigators gain a greater understanding of these different mechanisms of Bt resistance and perhaps discover more, this information could lead to improved resistance management and better decision making in the development of the next generation of biopesticides.

### 3.6. Role of Insect Immunity in Resistance

When a susceptible insect consumes plant tissue and, therefore, Cry and Vip families of insecticidal proteins, the hypothesized ultimate cause of death is sepsis caused by gut bacteria invading the body cavity. We hypothesized from this study that an additional potential mechanism of Bt-resistance may involve an enhanced immune system. We found three differentially expressed immune related pathways in the bollworm, JAK/STAT, Toll, and IMD. The primary function of the IMD pathway is to respond to infection by gram-negative bacteria. Major components of this pathway include, but are not limited to, Peptidoglycan recognition proteins (PGRPs), Peptidoglycan binding proteins, Fas-associated death domains (FADD), DREDD, Relish, Transforming Growth factors (TAK or TAB), Nuclear factors kappa beta (nf-kb), immunoglobulin binding proteins, Fas binding factors, caspases, and defensive proteins [35,36]. In this study, a number of transcripts (9) involved in the IMD pathway were found to have increased expression in the resistant strain of the bollworm. These were a Fas-binding factor (+3.75 log2 fold change), immunoglobulin binding protein (+3.41 log2 fold change), Peptidoglycan recognition protein C (+1.86 log2 fold change), Peptidoglycan binding protein (+1.43 log2 fold change), Nuclear factor kappa betas (+0.88, +0.74 log2 fold change), and transforming growth factor betas (+0.54, +0.48 log2 fold change). A recent study conducted by Liu et al. (2019) also correlated PGRP expression to Cry1Ac proteins [37]. This may indicate that the IMD pathway, in particular, PGRP proteins, are an important mechanism for Bt-resistance.

The Toll pathway is another immune-response pathway in *H. zea*. This pathway responds to gram-positive bacterial and fungal infections in addition to being involved in developmental processes. Some components of the Toll pathway are shared with the IMD pathway. Major protein components of the Toll pathway are Spatzle, Toll receptors, Beta-1,3, glucanases, Cactus proteins, Dorsal proteins, death associated protein kinases (DAP), and cecropin proteins [35,36]. We found that transcripts involved in the Toll pathway were found to have increased expression in the resistant strain. These included beta-1,3, glucanase (+1.47 log2 fold change), DAP kinases (+0.68 log2 fold change), Toll protein (+0.33 log2 fold change), and Toll receptor (+1.30 log2 fold change).

A third immune pathway in insects is JAK/STAT, and components of this pathway demonstrate differential expression between the resistant and susceptible bollworms studied. This pathway deals with more generalized immune responses and developmental processes, rather than specific types of bacterial infection. Major components of this pathway include Domeless receptors, Hopscotch kinases, STAT proteins, PIAS regulators, SOCS proteins, and defensive proteins [36]. We found differential expression levels in the following transcripts: Hopscotch kinase (+1.1 log2 fold change), STAT5B (+0.59 log2 fold change), SH3 binding protein (+0.68 log2 fold change), and PIAS3 (a negative regulator) (−0.79 log2 fold change).

Of the three immune pathways examined, the IMD pathway had the greatest number of transcripts with increased expression in the resistant strain (nine transcripts) as well as the highest degree of log2 fold change (Fas-binding factor (+3.75 log2 fold change), immunoglobulin binding protein (+3.41 log2 fold change), and Peptidoglycan recognition protein C (+1.86 log2 fold change)). This could potentially be explained by the fact that gram-negative bacteria commonly colonize gut-cavities of lepidopterans [38]. Because the recognized cause of death by Cry proteins is from sepsis when bacteria move from the gut to the hemocoel, this could explain why the IMD pathway saw the most differences in expression. It is important to note that both gram-negative and gram-positive bacteria are present in lepidopterans with gram-positive the most dominant [38].

The increased expression in these three immune pathways could be a mechanism of insecticide resistance through an enhanced ability to fight bacterial infection. This study provides some of the first insight into the possible role of the insect immune system in Bt-resistance. Previous research has found that the gut-microbiome and immune activity in insects is linked to chlorpyrifos resistance; potentially similar interactions are happening in Bt-resistance [38]. However, because this study only considered the correlation between gene expression and Bt-resistance, further investigation is needed in order to support the hypothesis that enhanced immunity has a role in insect resistance to transgenic crops.

In summary, this study has confirmed most of the previous work on identifying possible mechanisms for caterpillar Bt-resistance, but, in this case, all of these mechanisms appear to be in play in a single resistant strain, to an extent not shown before. In the resistant strain, we found increased expression of multiple proteases, transporters, tetraspanins, and secretases and decreased expression of Cry midgut receptors. Additional differential expression was found for enzymes typically involved in resistance to chemical insecticides. This could be a result of differences in a laboratory versus field strain or maybe a mechanism by which resistant caterpillars detoxify increasing levels of microbial metabolites in the hemocoel during the advancement of sepsis. Furthermore, there were clear changes in three of the insect’s major immune pathways that could provide an enhanced mechanism to fight infection from Bt induced sepsis. These pathways were characterized for the first time for bollworms. Further research is needed in order to confirm a role of the immune system in Bt resistance. While other studies have examined Bt-resistance in lepidopterans (Asian corn borer, *Ostrinia furnacalis*, diamondback moth, *Plutella xylostella*, and the old world bollworm, *Helicoverpa armigera* [47,48,49]) using RNAseq and lab-selected resistance, this study with *Helicoverpa zea* represents the first work that used RNAseq to examine expression levels in a field-caught resistant strain with no selection for resistance prior to sequencing. This study also supports the findings in these other Lepidoptera, including decreased expression levels for Bt-receptors as well as increased trypsin and other mid-gut proteases and increases in detoxifying/metabolic enzymes (P450s).

## 4. Materials and Methods

### 4.1. Sample Collection and Preparation

*Helicoverpa zea* Bt-resistant (Cry1Ac, 100-fold resistant; see results section) eggs were obtained from a colony that was established at NCSU. The susceptible insects were from a laboratory strain that was reared with no exposure to Bt for 18 years, while the resistant strain was originally collected from the field in NC and reared in the laboratory for two generations. The resistant colony was collected from Wake Forest, North Carolina, USA in non-Bt corn (2017). The susceptible colony was obtained from Benzon Research, Inc. (Carlisle, PA, USA). Both colonies were reared in the lab for two generation on artificial diet [30]. To eliminate strain differences as much as possible, both the resistant and susceptible bollworms were reared in the same laboratory at NCSU and on the same artificial diet, under the same environmental conditions, and using the same rearing methods. Rearing conditions were as follows in a growth chamber: 14:10 L:D, 27 °C:24 °C L:D, and 60% RH, and they were mated to conspecifics for each colony. The artificial diet was *H. zea* diet (Southland Products, Lake Village, AR, USA). The same rearing methods were used, as described in Reisig et al., for both resistant and susceptible colonies [30]. Neonates less than 6 h after hatching from both colonies were then used for diet-based susceptibility bioassays and also RNA extraction.

### 4.2. Cry1Ac Susceptibility Bioassays

For both bioassays, 128-well plastic trays (Bio-assay tray bio-ba-128, Frontier Agricultural Sciences, Newark, DE, USA) were used. The overlay method with Cry1Ac protein (94%–96% pure, trypsin activated, ion exchange HPLC purified, desalted, freeze dried, provided by Marriane Pusztai-Carey, Case Western Reserve University) dissolved in Triton X-100 buffer (0.1%) was used for toxin application. For each well, 200 µL of Cry1Ac was added in the following concentrations: 0 µg/cm^2^, 0.1 µg/cm^2^, 1 µg/cm^2^, 5 µg/cm^2^, 10 µg/cm^2^, 25 µg/cm^2^, and 100 µg/cm^2^. Each concentration was placed in 64 individual wells per bioassay tray where 1 neonate insect was placed using a fine tip paintbrush immediately after drying. In total, 128 neonates were used for the Bt-susceptible strain assays and 448 neonates for the Bt-resistant assays. The trays were then returned to the growth chamber for 7d. Mortality was then recorded, which was determined by whether or not neonates moved upon prodding with a brush. Data from each well and concentration of Cry1Ac were then pooled and used to calculate the LC_50_ and 95% CIs using a SAS probit analysis (PROC PROBIT, SAS Institute 2008). The OPTC and LOG10 options were used to model the responses. The same bioassay and LC_50_ calculation methods were used, as described in Reisig et al. [30].

### 4.3. RNA Extraction

From these colonies, five Bt-resistant samples and five Bt-susceptible samples were prepared, each of these samples made from 10 neonate *H. zea*. All of the neonates were lab-reared and unfed prior to RNA extraction as described earlier. Neonates were mechanically homogenized into one DNAse and RNAse free tube for each sample. From each sample, total RNA was extracted while using the RNeasy Mini Kit following the manufacturer’s protocol (Qiagen, Valencia, CA, USA). Purity of total RNA in each sample was then assessed using an Agilent 2100 Bioanalyzer (Agilent Technologies, Santa Clara, CA, USA) by the NC State University Genomics Core Facility (Raleigh, NC, USA). Sequencing was then only conducted on samples that had sufficient purities (RNA Integrity Number > 9.0).

### 4.4. RNA Sequencing

The NCSU Genomics Core Facility also conducted RNA-seq for this experiment. cDNA libraries for each sample (using 500 μg of total RNA each) were constructed to prepare for RNA-seq using the TruSeq RNA Library Prep Kit v2 (Illumina, San Diego, CA, USA) following the manufacturer’s protocol. Transcriptome sequencing was performed on the NextSeq 500 System (Illumina, San Diego, CA, USA) while using a paired end setting and read length of 2 × 150 base pairs. A sequencing depth of > 25 million reads per library was obtained using a High Output Flow Cell. A total of 10 mRNA libraries were then prepared, five each for resistant and susceptible. The SRA Toolkit v2.9.2 was used to convert raw reads to fastq files [50]. Fastq file read quality was then assessed using the FastQC tool v0.11.7 [51]. A Phred score of > 30 was required for a majority of the sequencing reads in order to establish a baseline for quality. Fastq files with appropriate quality then proceeded to assembly and quality control steps.

### 4.5. Transcript Assembly and Quality Control

The NC State Bioinformatics Core (Raleigh, NC, USA) conducted transcript assembly and quality control. The reads were assembled using the StringTie program (v1.3.5, John Hopkins University, Baltimore, MD, USA) with 45224 primary transcripts assembled into transcript set 1 with the *Helicoverpa zea* reference genome [52]. The program Trinity (v2.8.4, Broad Institute and Hebrew University of Jerusalem, Jerusalem, Israel) was used to assemble an alternate set of transcripts (set 2) that did not align with StringTie in order maximize transcript assemblies [53]. For transcript assembly, there were 149108 transcripts assembled and then processed through the Blobology program (v2.15.2, University of Edinburgh, Edinburgh, UK) in order to determine whether contaminants were present [54]. Transcripts that matched to Lepidoptera were then saved (108,867 transcripts). From these, all of the ribosomal RNA transcripts were deleted from this transcript set. The Evigene program (v1.0, University of Indiana, IN, USA) was then used to cluster the remaining 108,841 transcripts which resulted in 34,059 transcripts in set 1 [55]. Transcript sets 1 and 2 were then combined and clustered using Evigene, resulting in 26,800 primary and 12,095 alternate transcripts. Primary and alternate sets were then run through Blobology to check for contaminates once again. Ribosomal RNA transcripts were also removed from these sets. Primary and alternate transcripts were then clustered and combined with Evigene.

Fastq files for each replicate were trimmed for adapter sequence and quality using the TrimmoMatic sequence trimmer (v0.39, Max Planck Institute, Jülich, Germany) [56]. The *Helicoverpa zea* reference genome (NCBI), was used to map each trimmed file to the reference genome using HiSat2 [57]. StringTie was then used to assemble the resulting mapped files in order to assemble RNA-seq alignments into potential transcripts. All of the transcript annotations from each replicate were then merged into one “expressed transcriptome” file. This was then used to guide gene boundaries when calculating differential expression values (log2 fold change) between the susceptible and resistant strains via CuffDiff (v7.0, Cambridge, MA, USA) [58]. Statistical significance was determined while using the Tuxedo Pipeline (in CuffDiff, which assigned transcript *q*-values, α = 0.05). Only statistically significant transcripts were included in later data analysis [59]. These results were then imported into the R statistical software platform for quality control checks and visualization of results [60]. The sequence of transcripts that were determined to be differentially expressed were extracted from the reference genome and used in a BLAST search against insects in order to provide initial annotations. Quality control steps were conducted with FPKM, boxplots, MDS plot, PCA plot, normalization, heatmap, and volcano plots. All of the quality control steps were passed by all replicates. After all transcripts were assembled and quality control steps passed, 6098 transcripts were identified as differentially expressed in this experiment. Of these 6098, 3042 transcripts had higher expression in the susceptible strain with 267 being found only in this strain. The remaining 3056 had higher expression in the resistant strain with 323 only being expressed in the resistant strain. Blast2GO (v5.2.4) was implemented to functionally annotate open reading frame assignments [31]. Gene ID and function was determined using BLASTx (*E*-value cut off 1 × 10^−5^), using lepidopteran taxonomy to filter results, running against the nr and swissprot databases [31].

### 4.6. Data Analysis and Figure Construction

Figures and tables for this paper were constructed while using Microsoft Excel, PowerPoint, Word (2018), and SigmaPlot (v14.0, SigmaPlot, Systat Software, San Jose, CA, USA). Venn diagrams were constructed using Venny 2.1 (http://bioifogp.cnb.csic.es/tools/venny) in order to depict transcript differences between strains. OmicsBox (v1.3.3) (BLAST2GO) was also used to construct gene ontologies (https://www.biobam.com/omicsbox-apps/) [31].

## Figures and Tables

**Table 1:** Top 50 (highest log2 fold change) up-regulated transcripts in the Bt-resistant strain of the bollworm, *Helicoverpa zea*, including gene identity, general function, and magnitude of log2 fold change.

	Gene ^a^	Gene ID ^b^	General Function ^c^	Log2 Fold Increase ^d^
1	Hzea.19884	WD repeat-containing protein on Y chromosome-like	Bromodomain protein	+9.08472
2	Hzea.21942	BAC, pupae DNA	Pupal protein	+7.16661
3	Hzea.16694	BAC, pupae DNA	Pupal protein	+6.87974
4	Hzea.24399	allergen Api m 6-like	Serine Type Endopeptidase, protease inhibitor	+6.86176
5	Hzea.23153	BAC, pupae DNA	Pupal protein	+6.51622
6	Hzea.2403	KGHa033C10 carboxyl/choline esterase	Dietary detoxification, hormone/pheromone degradation, neurodevelopment	+6.17351
7	Hzea.15968	fatty acid binding protein	Binding and transfer of fatty acids	+5.93447
8	Hzea.27515	zonadhesin-like	Facilitates binding of sperm to egg	+5.87046
9	Hzea.2907	sphingolipid delta(4)-desaturase/C4-monooxygenase DES2	Degenerative spermatocyte protein	+5.83895
10	Hzea.2119	serine/threonine-protein kinase dyrk1	Phosphorylation of serines and threonines	+5.51915
11	Hzea.8474	sorbitol dehydrogenase-like	Sorbitol metabolism	+5.51262
12	Hzea.3274	E3 ubiquitin-protein ligase Siah1-like	Proteosome mediated protein degradation	+5.46839
13	Hzea.4257	trypsin 3A1-like	Intestinal protein degradation	+5.31623
14	Hzea.23538	transmembrane protease serine 9-like	Serine protein cleavage	+5.13044
15	Hzea.32418	zinc finger protein 266-like	DNA binding domain protein	+5.0701
16	Hzea.18297	heat shock protein Hsp-12.2-like	Cellular stress response protein	+5.02824
17	Hzea.18477	AY2 tetraspanin 1 (TSPAN1)	Protein stabilization, cell signaling pathways, associated with BT resistance	+4.91784
18	Hzea.13946	facilitated trehalose transporter Tret1	Trehalose transport from fat body	+4.73271
19	Hzea.10453	gamma-secretase subunit pen-2	Cleavage of transmembrane proteins	+4.70584
20	Hzea.31473	JP123 retrotransposon HaRT3	Replication	+4.6664
21	Hzea.16029	nose resistant to fluoxetine protein 6-like	Uptake of lipids and xenobiotics from intestines	+4.64037
22	Hzea.1396	BAC, pupae DNA	Pupal protein	+4.5819
23	Hzea.15497	chymotrypsin-1-like	Intestinal protein degradation	+4.44894
24	Hzea.14146	cytochrome P450 6B5-like	Xenobiotic metabolism	+4.4463
25	Hzea.8807	BAC, pupae DNA	Pupal protein	+4.43852
26	Hzea.20434	stabilizer of axonemal microtubules 2	Microtubule binding	+4.42253
27	Hzea.16622	deoxyribose-phosphate aldolase	Deoxyribose phosphate catalysis	+4.37083
28	Hzea.9448	cytochrome P450 337B2v2	Xenobiotic metabolism, associated with insecticide resistance	+4.23841
29	Hzea.31690	H/ACA ribonucleoprotein complex subunit 1-like	Ribosome biogenesis and telomere maintenance	+4.14773
30	Hzea.8806	protein lethal(2)essential for life-like	Embryonic development	+4.14618
31	Hzea.27511	JP151 retrotransposon HaRT2	Replication	+4.14218
32	Hzea.14487	BAC 79L08	Unknown	+4.13627
33	Hzea.15502	trypsin-like protease	Intestinal protein degradation	+4.12705
34	Hzea.29377	cytochrome P450 337B3v1	Xenobiotic metabolism, associated with insecticide resistance	+4.10688
35	Hzea.17522	JP151 retrotransposon HaRT2	Replication	+4.10241
36	Hzea.1891	odorant receptor Or1-like	Odorant receptor	+4.07168
37	Hzea.25785	BAC, pupae DNA	Pupal protein	+4.04936
38	Hzea.391	cytochrome P450 337B2v2	Xenobiotic metabolism, associated with insecticide resistance	+4.0049
39	Hzea.2400	trypsin-1-like	Intestinal protein degradation	+3.94767
40	Hzea.11080	neuroblastoma-amplified sequence-like	Vesicle binding/transport	+3.89792
41	Hzea.18643	H/ACA ribonucleoprotein complex subunit 1-like	Ribosome biogenesis and telomere maintenance	+3.89234
42	Hzea.30733	NAD-dependent protein deacylase sirtuin-5, mitochondrial-like	Protein Deacylation	+3.78646
43	Hzea.10806	BAC, pupae DNA	Pupal protein	+3.77303
44	Hzea.2665	BAC, pupae DNA	Pupal protein	+3.76731
45	Hzea.15446	fas-binding factor 1 homolog	Binding protein, cell stabilization	+3.75496
46	Hzea.19761	BAC, pupae DNA	Pupal protein	+3.67297
47	Hzea.12185	trypsin, alkaline C-like	Intestinal protein degradation	+3.62558
48	Hzea.23659	BAC, pupae DNA	Pupal protein	+3.57875
49	Hzea.29138	zinc finger protein OZF-like	DNA binding domain protein	+3.56187
50	Hzea.15307	pancreatic triacylglycerol lipase-like	Lipid metabolism	+3.50668

^a^. Gene number corresponds to sequence number in Fastq files. ^b^. Gene ID annotations were found from an NCBI BLAST search top result (query coverage, *E*-value, percent identity). ^c^. General function determined by NCBI and UniProt database searches. ^d^. Plus indicates increased expression in the resistant strain.

**Table 2:** Top 50 (highest level of log2 fold change) down-regulated transcripts in in the Bt-resistant strain of the bollworm, *Helicoverpa zea*, including gene identity, general function, and magnitude of log2 fold change.

	Gene ^a^	Gene ID ^b^	General Function ^c^	Log2 Fold Decrease ^d^
1	Hzea.11969	cytochrome P450 337B3v1	Xenobiotic metabolism, associated with insecticide resistance	−7.18074
2	Hzea.27562	family 31 glucosidase KIAA1161-like	Glucose metabolism	−6.66764
3	Hzea.17125	protein ALP1-like	Cytoskeletal development	−6.26055
4	Hzea.29678	tudor domain-containing protein 7A	Post-transcriptional modification	−5.60295
5	Hzea.812	cytochrome P450 337B3v1	Xenobiotic metabolism, associated with insecticide resistance	−5.56349
6	Hzea.6938	splicing factor 3B subunit 4-like	Gene Splicing	−5.09605
7	Hzea.12845	putative nuclease HARBI1	Nucleic Acid cleavage	−5.01358
8	Hzea.31102	cuticle protein-like	Cuticle structural protein	−4.96903
9	Hzea.15432	transcription factor Adf-1-like	Adh gene expression regulation	−4.93912
10	Hzea.28236	ATP-binding cassette sub-family G member 1	ABC transporter protein	−4.92069
11	Hzea.14683	BAC, pupae DNA	Pupal Protein	−4.82383
12	Hzea.12475	isolate AD2 clone 1 microsatellite D47	Unknown	−4.78341
13	Hzea.7219	BAC, egg DNA	Oval DNA	−4.77338
14	Hzea.4118	multiple inositol polyphosphate phosphatase 1-like	Regulates cellular inositol levels	−4.63011
15	Hzea.28234	serine/threonine-protein kinase RIO1	Ribosomal subunit maturation	−4.61965
16	Hzea.15388	zinc finger protein 628-like	DNA binding domain protein	−4.61078
17	Hzea.13573	glucose dehydrogenase [FAD, quinone]-like	Glucose metabolism	−4.56858
18	Hzea.811	BAC 33J17 cytochrome P450 337B3v1	Xenobiotic metabolism, associated with insecticide resistance	−4.54212
19	Hzea.17013	small nuclear ribonucleoprotein F	Pre-mRNA splicing	−4.54025
20	Hzea.32168	UDP-glucuronosyltransferase 2B4-like	Glucuronidation catalysis	−4.51753
21	Hzea.4237	KGHa033C10 carboxyl/choline esterase CCE001f	Dietary detoxification, hormone/pheromone degradation, neurodevelopment	−4.4949
22	Hzea.21278	facilitated trehalose transporter Tret1-like	Trehalose transport from fat body	−4.46546
23	Hzea.25419	putative nuclease HARBI1	Nucleic Acid cleavage	−4.41916
24	Hzea.9745	BAC 18J13 cytochrome P450 337B2v2 and cytochrome P450 337B1v1	Xenobiotic metabolism, associated with insecticide resistance	−4.32618
25	Hzea.13850	BAC, pupae DNA	Pupal Protein	−4.30511
26	Hzea.12890	fatty acid synthase-like	Fatty acid synthesis	−4.27779
27	Hzea.4150	phospholipase A2-like	Fatty acid cleavage	−4.24604
28	Hzea.3034	BAC, pupae DNA	Pupal Protein	−4.20592
29	Hzea.13370	cytochrome P450 6B6	Xenobiotic metabolism	−4.19982
30	Hzea.11776	cuticle protein 65	Cuticle structural protein	−4.10268
31	Hzea.4423	lipase member K-like	Lipid metabolism	−3.98569
32	Hzea.23288	cuticle protein 1	Cuticle structural protein	−3.97585
33	Hzea.25418	putative nuclease HARBI1	Nucleic Acid cleavage	−3.95527
34	Hzea.6856	enoyl-CoA delta isomerase 1, mitochondrial-like	Fatty acid oxidation	−3.95465
35	Hzea.4455	dihydrofolate reductase	Dihydrofolic reduction	−3.931
36	Hzea.21115	zinc finger BED domain-containing protein 1-like	DNA binding domain protein	−3.9258
37	Hzea.14952	guanine nucleotide-binding protein G(q) subunit alpha	Guanine binding	−3.8824
38	Hzea.20452	cytochrome P450 337B2v2	Xenobiotic metabolism, associated with insecticide resistance	−3.8696
39	Hzea.13504	protein deltex	Cell communication, Notch pathway	−3.86954
40	Hzea.11171	organic cation transporter protein-like	Transport protein, cations	−3.81687
41	Hzea.14992	alkaline phosphatase 2	BT receptor in intestines	−3.77113
42	Hzea.30431	BAC, pupae DNA	Pupal Protein	−3.75267
43	Hzea.7746	cytochrome P450 337B2v2	Xenobiotic metabolism, associated with insecticide resistance	−3.71829
44	Hzea.5603	UDP-glucuronosyltransferase 2B7-like	Glucuronidation catalysis	−3.71518
45	Hzea.21691	BAC, pupae DNA	Pupal Protein	−3.69295
46	Hzea.17647	beta-secretase 1-like	Protein Cleavage	−3.64879
47	Hzea.32280	tyrosine--tRNA ligase	Ligation of tRNA and tyrosine, translation	−3.6039
48	Hzea.23371	CD209 antigen-like protein 2	Pathogen recognition receptor	−3.59733
49	Hzea.14510	BAC, pupae DNA	Pupal Protein	−3.5325
50	Hzea.21503	polyribonucleotide nucleotidyltransferase 1	Transferase protein	−3.48953

^a^. Gene number corresponds to sequence number in Fastq files. ^b^. Gene ID annotations were found from an NCBI BLAST search top result (query coverage, *E*-value, percent identity). ^c^. General function determined by NCBI and UniProt database searches. ^d^. minus indicates decreased expression.

**Table 3:** Highly up-regulated (threshold log2 fold change ≥2.0) *Helicoverpa zea* transcripts associated with insecticide or Bt-resistance (categorized by gene family and organized by function) including gene identity, general function, and magnitude of log2 fold change.

	Gene ^a^	Gene ID ^b^	General Function ^c^	Log2 Fold Change ^d^
**Tetraspanins**	Hzea.18477	Tetraspanin 1	Protein stabilization, cell signaling pathways, associated with BT resistance	+4.92
	Hzea.11255	Tetraspanin 1	Protein stabilization, cell signaling pathways, associated with BT resistance	+3.37
	Hzea.498	Tetraspanin 1	Protein stabilization, cell signaling pathways, associated with BT resistance	+2.88
**Serine Proteases**	Hzea.23538	Serine Protease 9-like	Protein cleavage	+5.13
	Hzea.24399	allergen Api m 6-like	Serine Type Endopeptidase	+6.86176
	Hzea.7824	Serine Protease Snake-like	Protein cleavage	+3.1
	Hzea.5128	Serine Protease 9-like	Protein cleavage	+2.4
	Hzea.16515	Serine Protease 3-like	Protein cleavage	+2.23
**Secretase Proteins**	Hzea.10453	Gamma-Secretase	Cleavage of transmembrane proteins	+4.7
	Hzea.30068	Beta-Secretase 1-like	Protein Cleavage	+3.04
	Hzea.27773	Beta-Secretase 1-like	Protein cleavage	+2.07
**Trypsin Proteins**	Hzea.15497	Chymotrypsin 1-like	Intestinal protein cleavage	+4.44
	Hzea.4257	Trypsin 3A1-like	Intestinal protein cleavage	+5.31
	Hzea.15502	Trypsin-like Protease	Intestinal protein cleavage	+4.12
	Hzea.2400	trypsin 1-like	Intestinal protein cleavage	+3.94
	Hzea.12185	Trypsin, Alkaline C like	Intestinal protein cleavage	+3.62
	Hzea.12186	Trypsin, Alkaline C like	Intestinal protein cleavage	+2.17
**Bt-Receptors**	Hzea.14992	Alkaline Phosphatase 2	Intestinal receptor for Cry1Ac	−3.77
	Hzea.17125	Alkaline Phosphatase 1 like	Intestinal receptor for Cry1Ac	−6.26
	Hzea.11178	Mutant Cadherin (BtR)	Intestinal receptor for Cry1Ac	+2.93
	Hzea.18127	Cadherin-like	Intestinal receptor for Cry1Ac	−2.29
	Hzea.20	Caherin-r15	Intestinal receptor for Cry1Ac	−2.89
	Hzea.8058	Aminopeptidase 1D	Intestinal receptor for Cry1Ac	−2.02
	Hzea.16397	Aminopeptidase 1	Intestinal receptor for Cry1Ac	−2.21
**Peptidases**	Hzea.12825	Carboxypeptidase B like	Peptide cleavage	+3.04
	Hzea.7667	Carboxypeptidase B like	Peptide cleavage	−2.03
**Transporters**	Hzea.29517	Multidrug Resistance protein 1 like	Efflux transporter	+2.22
	Hzea.3344	Multidrug Resistance protein 4 like	Efflux transporter	−2.06
	Hzea.9148	ABC Transporter ABCC3	Transporter protein	+3.36
	Hzea.10318	ABC Transporter ABCC3	Transporter protein	+3.06
	Hzea.13698	ABC Transporter ABCC2	Transporter protein	+2.6
	Hzea.13541	ABC Transporter ABCC3	Transporter protein	+2.5
**Chitin**	Hzea.21994	Chitin Synthase A & B	Chitin synthesis	+3.11
	Hzea.18297	Heat Shock Protein 12.2	Cellular stress response	+5.02
**Metabolic transcripts**	Hzea.9448	CYP337B2v2 & CYP337B1v1	Xenobiotic metabolism, associated with resistance to insecticides	+4.23
	Hzea.29377	CYP337B3v1	Xenobiotic metabolism, associated with resistance to insecticides	+4.1
	Hzea.391	CYP337B2v2 & CYP337B1v1	Xenobiotic metabolism, associated with resistance to insecticides	+4
	Hzea.20633	CYP337B3v1	Xenobiotic metabolism, associated with resistance to insecticides	+3.37
	Hzea.6595	CYP367A2	Xenobiotic metabolism	+2.43
	Hzea.1468	CYP4AU1	Xenobiotic metabolism	+2.31
	Hzea.13660	CYP337B3v1	Xenobiotic metabolism, associated with resistance to insecticides	+2.22
	Hzea.18411	CYP421A5	Xenobiotic metabolism	+2.16
	Hzea.16306	CYP337B3v1	Xenobiotic metabolism, associated with resistance to insecticides	+2.04
	Hzea.29950	CYP337B3v1	Xenobiotic metabolism, associated with resistance to insecticides	+2.01
	Hzea.2403	carboxyl/choline esterase CCE001f	Xenobiotic metabolism, associated with resistance to insecticides	+6.17351
**Protease**	Hzea.3274	E3 ubiquitin-protein ligase Siah1-like	Proteosome mediated protein degradation	+5.46839

^a^. Gene number corresponds to sequence number in Fastq files. ^b^. Gene ID annotations were found from an NCBI BLAST search top result (query coverage, *E*-value, percent identity). ^c^. General function determined by NCBI and UniProt database searches. ^d^. Plus indicates increased expression in the resistant strain, and minus indicates decreased expression.

**Table 4:** *Helicoverpa zea* differentially expressed transcripts associated with immune functions (organized by immune pathway or general function) including gene identity, strain of bollworm, general function, and magnitude of log2 fold change.

Gene ^a^	Gene ID ^b^	Strain	General Function ^c^	Log2 Fold Change ^d^
Hzea.27139	Peptidoglycan (PGN) Recognition Protein C	Resistant	IMD immune pathway	+1.86
Hzea.24180	lysM & PGN binding protein 2	Resistant	IMD immune pathway	+1.43
Hzea.15134	PGN recognition protein LB	Resistant	IMD immune pathway	+1.11
Hzea.8213	NF-kappa-beta p110 subunit	Resistant	IMD immune pathway	+0.88
Hzea.3860	NF-kappa-beta binding protein	Resistant	IMD immune pathway	+0.74
Hzea.11065	Immunoglobulin binding protein	Resistant	IMD immune pathway	+3.41
Hzea.15446	Fas-binding factor 1	Resistant	IMD immune pathway	+3.75496
Hzea.18171	Transforming Growth Factor Beta 1	Resistant	IMD immune pathway	+0.545215
Hzea.22952	Transforming Growth Factor Beta 1 Receptor	Resistant	IMD immune pathway	+0.486983
Hzea.12555	TGF-Beta Activated Kinase 1	Susceptible	IMD immune pathway	−0.584251
Hzea.2642	Cecropin 1	Susceptible	Antibacterial protein	−3.39285
Hzea.5285	Lysozyme-like	Resistant	Antimicrobial protein	+0.984174
Hzea.8918	Lysozyme-like	Susceptible	Antimicrobial protein	−0.469066
Hzea.5268	Lysozyme	Susceptible	Antimicrobial protein	−3.34181
Hzea.19610	Putative Defense Protein Hdd11	Resistant	Pathogen Defense	+1.11573
Hzea.28790	Beta-1,3-glucanase protein	Resistant	Pathogen recognition protein	+1.47469
Hzea.30418	Nodulin 75 like	Resistant	General Immune response	+1.55155
Hzea.5226	Adaptor molecule Crk	Resistant	Immune signaling	+0.598283
Hzea.5227	Adaptor molecule Crk	Susceptible	Immune signaling	−0.79207
Hzea.8567	Autophagy protein 5	Resistant	Programmed cell death, removes unnecessary cells. Involved in immunity	+0.984792
Hzea.23318	Autophagy Related protein 16-1	Resistant	Programmed cell death, removes unnecessary cells. Involved in immunity	+0.918917
Hzea.21156	Autophagy Related protein 13	Resistant	Programmed cell death, removes unnecessary cells. Involved in immunity	+0.80002
Hzea.25537	Autophagy Related protein 2 homolog A	Susceptible	Programmed cell death, removes unnecessary cells. Involved in immunity	−0.690981
Hzea.25536	Autophagy related protein 2 homolog A	Susceptible	Programmed cell death, removes unnecessary cells. Involved in immunity	−1.00944
Hzea.25507	Autophagy related protein 2 homolog A	Only in Resistant	Programmed cell death, removes unnecessary cells. Involved in immunity	N/A
Hzea.2633	C-type lectin	Susceptible	Immune response to pathogens	−1.07
Hzea.19146	Death associated protein kinase 1	Resistant	Toll immune pathway	+0.678001
Hzea.15107	Toll like receptor 3	Resistant	Toll immune pathway	+1.29648
Hzea.4906	Toll like protein	Resistant	Toll immune pathway	+0.327495
Hzea.17194	Signaling intermediate in Toll pathway	Susceptible	Toll immune pathway	−0.83
Hzea.24906	CD63 Antigen	Resistant	General Immune response	+1.08759
Hzea.23769	Antigen 8	Resistant	General Immune response	+0.582441
Hzea.25130	CD109 antigen like	Resistant	General Immune response	+0.529484
Hzea.24900	CD63 antigen like	Resistant	General Immune response	+0.513073
Hzea.25569	H13 Antigen	Resistant	General Immune response	+0.334133
Hzea.24905	Antigen CD53 like	Susceptible	General Immune response	−0.8215
Hzea.8177	cell nuclear antigen	Susceptible	General Immune response	−1.15965
Hzea.23371	CD209 antigen like protein 2	Susceptible	General Immune response	−3.59733
Hzea.9589	CD63 antigen	Only in Susceptible	General Immune response	N/A
Hzea.11558	CD109 antigen	Susceptible	General Immune response	−1.03885
Hzea.8646	tyrosine kinase hopscotch	Resistant	JAK/STAT pathway	+1.10303
Hzea.19419	signal transducer and activator of transcription 5B (STAT5B)	Resistant	JAK/STAT pathway	+0.589054
Hzea.17134	SH3 domain-containing kinase-binding protein 1	Resistant	JAK/STAT pathway	+0.676851
Hzea.3426	E3 SUMO-protein ligase PIAS3	Susceptible	JAK/STAT pathway	−0.79

^a^. Gene number corresponds to sequence number in Fastq files. ^b^. Gene ID annotations were found from an NCBI BLAST search top result (query coverage, *E*-value, percent identity). ^c^. General function determined by NCBI and UniProt database searches. ^d^. Plus indicates increased expression in the resistant strain, and minus indicates decreased expression.

## Supplementary Materials

The following are available online at https://www.mdpi.com/1422-0067/21/18/6528/s1, Figure S1 (A–J): Nucleotide sequences in the Bt resistant strain of *Helicoverpa zea* for unknown (not found in GenBank) or uncharacterized (found in GenBank but with no functional assignment) up regulated transcripts with the greatest log2 fold change., Figure S2 (A–J): Nucleotide sequences in the Bt resistant strain of *Helicoverpa zea* for unknown (not found in GenBank) or uncharacterized (found in GenBank but with no functional assignment) down regulated transcripts with the greatest log2 fold change.

## Abbreviations

ABC	*ATP-binding cassette transporter*
Bt	*Bacillus thuringiensis*
CYP	Cytochrome P450
GMO	Genetically modified organism
*H. zea*	*Helicoverpa zea*
IMD	Immune deficiency
PGRP	Peptidoglycan receptor protein
TSPAN	Tetraspanin
