# Supplementary material for: Multiple Known Mechanisms and a Possible Role of an Enhanced Immune System in Bt-Resistance in a Field Population of the Bollworm, Helicoverpa zea: Differences in Gene Expression with RNAseq
Source: Int J Mol Sci. 2020 Sep 7;21(18):6528. doi: 10.3390/ijms21186528 (PMC7555151; doi:10.3390/ijms21186528)
Supplement: Supplementary file 1 [file ijms-21-06528-s001.pdf]

Figure S1 (A-J). Nucleotide sequences in the Bt resistant strain of *Helicoverpa zea* for unknown (not found in GenBank) or uncharacterized (found in GenBank but with no functional assignment) up regulated transcripts with the greatest log2 fold change. Included left to right/top to bottom are gene ID, top BLAST result match, magnitude of log2 fold change, and nucleotide sequence 5′ to 3′. Lower case letters are tandem repeats. Additional sequence information available upon request to corresponding author.

(A).

>Hzea.1588.1 gene=Hzea.1588. “Unknown Gene” Log2 fold change= 9.52

GAGTCGACTAGTTTCAAAGTCGGCAATCtgactttagtatttttttggttatgTCAAAAAAACGCCTTACTTGGT
GTTAGTTGTCTTTAGGATTCCAAAACTacttttgaaattttctttaatattcaaCAGAACTAATTAGTCAtggtttttatattt
ctttactaaGAATaagtcataaattaaatattaattttgcagaTTGCCGGATatgaaattaagttattaagATAGCCGGGTTTGAAA
CTGACATATGTGCAGATTGCCATTTTTGATACCAAATACCTGCTTATAAAATCAGGGTTGTCACA
ATAGCATAAAAATTATGCTGTCTGACTGAAAGTATGGGGGTATATTTAAGGggacttataaataaaaaatgg
gcTCATAAAATCAAACTTTTAATTGCACTTACTTCTTACacaaattttaaaagaatattattatatacggAGATCTGG
AACCTCTTCTAACGCCTCATCGCAATAATCAGAAGGTGGTATAGTCGATGCATCAAGCGACTCCT
GctcatcaaaaatattacaataatatgacAAAGTTGGAATTTCTTTCCACTCAGCACCAAAATGTTTGCACAACAG
CTTCTTCAAatctgataatttatttatctttattactaATTTTTTCATTTGCGACATTGTTAGAGTATCACACTTAAGC
GCCTTAGAGTTATGGTTACAAACTTTTTCTGGCATTGATATTGGGGTATATCTAAAAAGAATAGA
AAATACGTAAGCAGGTAAGTTAGCAGTAAGCACTTCAATAAAACCAAAGTTAACCCAAACAGC
ATGCCAGCTCGTGACAGCCCTAACTTCAAACTAACCTCAATATTTTAGCTTGGTTTCTTGACCAAT
TTTCGGGATCTCGTTTGCGTTTTCTTGCAAATTTATTAGTAACAAGGCTGGGGCTTGACTCCATTAT
AACTTTACACCTTCAATCacttaattgtaattaaaataacactaaaaacGTGTTAAAATCTCCGTACAATAATGTGC
GAGCGGCCATTACCAACGCTCGCTTCTGATTGGTCGATGACCTAAGATTGccgtttttgaataaaaacgtATT
AAAGAATGCCACATATGATCCTAAGCCAGgattttgattagatttttataTAGCAGATTACCGCTTTTGTTAGG
TAGCTTCTAATTCAAGcggttttattcaaatttatcTGTGTTACCACATTAATAActcaatttttgaaaaatgcaCAGATTG
CCGTCTTTGAAACTAGTCGACTCA

(B).

>Hzea.6945.1 gene=Hzea.6945 “Uncharacterized LOC110376017” Log2 fold change= 7.43

ttaaaactaagtGTTGACACGAGTAGGCGgtgtcgtgttgcactgcgtggtgtcACGTAGTCTCCCGCGGAATCGCCG
GAAAACGACACCTTTCGGCCAAAACTGTTCTTGGAACATGTCGAGATGCTGAGTCGGCACACGC
ACCACAAACGAATTAAAATTCGTGTTGTGTCTCGGCTCTAACTTCTCGACTCTCAATGTCCAGTTG
GTCTTCACACGTATATACTCCACGATCTCCTCGACCTTCGTGGAGTGATGCAGACGCGAAACATA
CAGCTGCGTCGTAGGTATTGCGGGACGCAACAGCATGTTCGGACCCGTCAATGCAGTTCCGCATC
GGTTTCGACTAGATGGCTTCTTCTTTATCCTCTCCACCTTGATGAAACCATCTGCATCAGCCGTCC
CTTTGCCAGGTTCAAGATGAACCCAGCCTTGGGTTACTTCGGGGAGACTCTGGGCTCCCACAATA
GCCTTGGTTAGCCGTTGAACGGGATCTTTCTTTACGGCAGCCGAGTAAACACGTCTAGGCTTCGA
CGTGCTTGCATTTATCGGCGTCCCGACGGCAGGAGACGCGGAGGGGGCGGGGCACAGTTTAGCA
TCACTAGCAGTGGCGACCGGCGACGAGTGCAATTTTGCTGACGCGGCGCTCAGCGGCGATGCGT
TGGCCTGTCCGTGGCGTGTGTTGTCGCTGTTGGCAGGCGCGTGTGACCTACATTCACAGTTACGTA
ATGCTGCTACTTCACTGCGTAGATCACTGATGGTAGATTGCGATGCTTCCAGCTTCAACTGCAGCT
CGACCAGGCTAGACCTCATACTCGTGATGTCTTTTAGGAGCCTGGTGACGTCGACGTGGTCAAAC
GTGACCGGGGGCAGCTTACGCAACTCCTTTGCCACGAAAGTTGGTACGTCGTCTGGGTCGGTCTC
CTTCAATACTTTAATTATATCTTGGAGACTCCTCTCTCCCttttcct

(C).

>Hzea.31522.1 gene=Hzea.31522 "Uncharacterized LOC110369980" Log2 fold change= 6.57

CCTcttttatggtaaatcgtaggcattttatggaccagggtcgcggtaactctttcgaacggACGGGGGTGACGTCACGGACGCCA
AGTTAGCCTGTGCTGGTTGCTGTTGTTTagcatcaaataaattaaaaaacgtaTACAATAGTAagaattatattacaaaaag
tcGAAGCTTAACCTAGTGCATCACATAAAACGTTATCTAGTGTTTTAGTGCAGTGTATTATAtgcaaaa
cttaaaactatttacaacaAAACTTAAAGCTAATTAACAGTGACCTAAATCTAccctaaacataaaaatataaacattaatcat
taaaaattatttgaaactaaGTGCTGAAGTGATACCAAAGTGCGCATATTACTCTGTTTACCCCAGTGTCGAT
ACATGGTGTCAGTAAACCATAGGACTCCCATCTAACCATAGCCGCACGCAAAGTTCTGAACAGC
GCCATCTTTTGATACTCAAAAGAATTTGTATCGATTGCTCCTGCATTTCGACACAGTCATGCCTGC
ATCTAGGTGTGGTGGTTGCAAGAAATATATGACGTCGATAGATGCCGCTACATGCATCAAATGCA
ATCGCATACACCATCGAGCTTGCGTTAGTCTGCCTGCAACGGGTTGTATTCCAACGAACTGGTGTT
GTCCGGAATGCCAGAAAAACCAGGTTAGGGACAATAAGGCGGAGACACCCGTAAGAGGAATGG
CAAGTTCTGTCCTTCCAAGGGAGCAGCTCTCTAAGGAGCTGGTGGATACCAGCCCGTTTAATTGT
ACTGCGCTGGACGAAACTACAACTTTCTCCCCGGATTTGGCTGCTGAACTTCGGGTGTTCAAGGA
GGAACTTCGTGCAGAGTTTCGCCTCATGCACAAAGAATTTCTTCAATTGCGGACAGAAGTAGCGC
AGCTAAAAGACGCCCTAAATAACTCTGGCGAGCGTATGGACGTGATCGAGGCGCGGGTTGAAGC
TCTCGAGCTGAAGTTCGAGCAGAAAATCCTGCCGACCAGCAACAGCGATTACGTCGACAACACT
ATCGCTGAATTGAAGTGCCAGCTTAACGAGCGTGATCAAGAACTGTTGCTCAACGACATTGAGAT
CACAGGAATCCCCGAGAGTAAGGAAGAAAGCACCCTCCACCTCGTGAACGTTCTCGCAGTAAAG
CTCGGCATTACACTAGACGAGAGGGACGTCGTCCATGCCGAACGGGTTGGAGCTACGCACCGCA
ACCGTGTTGTTGCCTTGGATGGCAGTATTGCTGCTGACCCCGCTGCGCAACGGCCGCGGAGTATA
TCGGTGCGCCTGGCAAGGCGCGCGACCCGCGACGCGCTACTGCGCGCGGCCCGAGTGCGTCGTG
GCTTGAGTACGGATGCACTCGATATGCCTGGCCCGCCGCACCGTGTTTACGTCAACGAGCGCCTC
ACGCGCACCAATCGCCGGTTATTTTATCGCGCCCGCCAGGCAGCGAGTCTGCATGGCTGGAAGTA
TGTGTGGACAAGGGAGGGTAAAATATTGTGTCGCAAGGACGATCGGTTAAAAGTAGAAGTTATT
AGACATGAAGACGATGCTACCAAAATTTTTGGCTAAAGATCTGTTTGAGGCTTAAGTTGTGCTTA
ATTGTTGTTGTCGTGCGTATGATTTTTCTTTGTTCTgtctttttattgttatatttgttttcgtgtttatttttattacttaattttatttgtc
ttataCATTCACttttaaacttgtttttctTCCTAATAAGTGTTCAGACCAAATGGTGCGCATTTCTTGCACTATC
GAATGGGAGTCCTCTTGGATTAATTGTTATCAACCCACTTTTA

(D).

>Hzea.6635.1 gene=Hzea.6635 "Unknown Gene" Log2 fold change= 6.22

NNNNNTTTGTTTTTAGCATTGATTCAGCCGTGTGAGAGGACGAGTTAAGTGATTTTGTTACCTCTA
CCGGGATGTTTGAGAAATACGAATCAAACGCGTTGGCAACTTCTGCTTTATCGGAGACgataacattatt
aataattaatcgGGGATCATTGTCACGTGATTTAGTTTTACCTGTTTCAGAGTTTATAATACTCCagacagtttt
tattttattgtcagatTTCGCAATTTTTCTACTAAGTGATTTGGATTTAGCAAGGGTACATACttttttgaatatctttga
GTAGTTCCTTACATAGTTATGGAAAGAAGGATCAGAAGTCATTGATTTTTCGTGGTATAAGTTGTA
TAGGACATCTCTACTTATTCTTATTCCTTTGGTAGACCAATCGCAAAATTTAAGTTTCTGTGaaattgta
actttttttttaggatatgatttattgaattcttgacataatttatggaacaagttattaaaattagtatttgGGTTGGAGGAATTCAAGCTAAT
ATTAGGCAATTTGTTTTCcacattattttgaatttattcaaagttgATTTTGTCAATGGTCtacaagtaattaatttatgtaaaacgg
gtttttcatttgttttataattaataagctGTCCTAAATGGTCTGACCTTACTCCAGATATTAGTGCAGAACTCTGAA
ATTCAGAGTTGGTAAAGATATTATCTATGCAAGAGCTTGAAGTAGCAGTTATTCTAGTTggttcattaaa
aatatgttttaaattaaatgatttaaataaatctaatagtTTACATTTCTCAGGTGACTCAGtaagaatatttatgttaaagtCCCCTGA
GACAAATACCAACTTGTTACTTTTACAAACTTTACTCAATATGTCCTCCATACAGtctaaaaatgatgaaaa
atttGATGTTGGAGGTCTGTAAAC

(E).

>Hzea.5167.1 gene=Hzea.5167 “Unknown Gene” Log2 fold change= 5.5

ACgggtttttcatttgttttataattaataagctGTCCTAAATGGTCTGACCTTACTCCAGATATTAGTGCAGAACTCT
GAAATTCAGAGTTGGTAAAGATATTATCTATGCAAGAGCTTGAAATAGCAGTTATTCTAGTTggttc
attaaaaatatgttttaaattaaatgatttaaataaatctaatagtTTACATTTCTCAGGTGACTCAGtaagaatatttatgttaaagtCCC
CTGAGACAAAGACCAACTTGTTACTTTTACAAACTTTACTCAATATGTCCTCCATACAGtctaaaaatga
tgaaaaatttGATGTTGGAGGTCTGTaaacacataaaattatatggCTTCCAATTTCTGCACAAGCTACCTCACACA
AGCGATCAACCGAGCCGGAAGTAATATCCTTTCTatctttaaattttaaattatttctacacAATATAATTACACCT
CCGCCTTCAGCTGACGCCCTGTTATAAACACCAGcaactacaaaattattaatactgAATAACATTTTCTCAGG
TTTCAACCAAGTCTCAGAAAAACACAATACATCTGTGGAGTACTTATCTATTAAAAGATCAATAT
CTAAAATTTTGCCAGAaaagcggtttatattttgatgGAAAAGTAGGATGTTATTAGTTCGCTTTTCTATTGTC
GGTGCACCTAGTTTAAATCTACCACAGTATTGTTGCAAAAGCTAGACAATACTGTATCAGTAGTT
TCCCTAAACTTTGTCATATCATTCTGTATGACCATGTTAATATTAAATGCTAATAGATTAgcaattttatc
tttatatttattgggATAAAACATAGTTTCCCgcgttaaataaaattctctaacaaaattgttagtatcaAAAAGCAATAATTTGT
CACTATGACAACATGTCAGGGTGTGAATTAAGCTGTTCAAGctatgaatataattattttgttctgtcGTGTAAGA
ATCTGAGTACGGGAGTGTACCtatcataatgtttttaaaattaagttttaacaaGGTGTTAATACCATTTACTAtgtcacttt
taattaaatcaaagctATTACCTATAAACAGTATCAGTGACGTATCATCATCAAATTGTTTgttgacaatattatttatt
ataaaattaaaatgcatgttTGGGTAACTATAACTACTAACACTACTTGTTGGCGAGAGGTAGtgt

(F).

>Hzea.908.1 gene=Hzea.908 “Unknown Gene” Log2 fold change= 5.12

AAACTATTTACAATCATAGTTTTCTGACTAACCAGAACATTAAAACCTTCCAAGTATTGCCTGggaa
aaattacatcaaatcaTGGTCCAACATTCGTTGCATGGATTAGGAAAACCCAGTAAAACCTAATCAGACcat
aaaataatcaatcataaatatcaaaataaaataacatgaattcaacataaaatacaacacaacaatacaacaataactcaaaaacattataaacataaattca
acataaaatacaacacAACAATTCAAcaataactcaaaaaaaaaaaacattattaacttaaaccCATCTTATCTCTGAACTGTCT
AAAAACAACAGCAGGCAGTGGCTTGGTTAATATATCGGCTATCTGTTCACCTCGCATCAGTATTT
TATCGTCCTTACTGATATACGTTCCACGCTGATTGAAGGTGATGGTCAGCCCAGCACGTTGCATCC
TCGACACCGATAGCAGGTTGTGTGGCGCCTCAGGACAGAACAACACGTCCTCCAAAGTGCCCTG
GATGCCTGTGTCGCTCATCACTTCCAGTGTTCCACGTTTGGTGGctgtaattaatatttcttcaaGGAAGGTATA
ACGTCTGCCTCCTAACACAGATGAAAAGAAAAAG

(G).

>Hzea.26512.1 gene=Hzea.26512 “Uncharacterized LOC111349884” Log2 fold change = 5.06

TTGAAAATTTAGATTTCTGTCTATAACAACACCAAGGTATCTAATAGAGTCCACTTTTTCAAGTTT
AGGGCATGTACAGTTGTTTGATGAATGATTATTACAAGAGTGGGCGATTATACCAAGAGCGGAA
TTCGATAGGAAGCGGGTATTTGTTAGTGTGAATGTGATATACTTTGTTTTGGTAGTATTTAATGTG
AGACGGTTGTTATTTAGCCAGCTTCCCACCACAGAAAACCCCTTTTCAGCATGGCTATAGACTTCT
TCCCATGTATCTCCATGAAAGAGTAGTACTGTATCATCAGCAAAAGTGATGATCTTTCCGTTAGTT
AGATTTAATTGGCACAGGTCATTTATGAACGTTAAAAATAGGGTTGGTCCAAGAATACTTCCCTG
CGGGACACCAATATTGACATTGCAATCATCACTTTGCCATCCCTCGACTATTACACGCTGATATCT
ATCACTAAGATAGCTCTGCAATAAATCCAGTTGGATTCCGCGTACGCCTACGTGTTCCAGTTTCTT
GAGCAAGAGTGGTGCCGAGACTGTGTCAAAAGCCTTTGCTAGGTCCAAAAATATTGCAAggactttttt
gttttcatcaaGTTTAGTTATAATAGTATCAGTTAATTGGTGAACTGCATCAGAAGTTGATTTACCTCTCC
TAAAACCGTATTGGTTtcagataatatatttttggattctaagtattttattagCTGTATGTTGATAGCTTTTTCTATAATT
CTAGATAAAACTGGCAAAATTGAAATTGGACGATAGTTATTGACACAGTCCCTGCTGCCACTCTT

GTAAATTGGATGAACCAAGGATTTTTAAGCTCCGCAGGGAAAACGCCTGTAGAGAAGCAAATG
TTGCAAATATATGCTATCGGCTGCGCTAAGATGTTAGCATATCTCTTAATGATACCACTAGGAAT
ATCGTTAAGTCCCTGAGCACAGCTGGTTTTAAGAGAGAGtataatagtttttatctcACTGGCATCAACTGGC
AGAAGTACAAAAGAAGAATCACGAGGAGGTAGGATGGGATATTGATTGTAGTTAGACAACCCT
AAAGTGGAGCCGGCCAATGAAGAACCTATGTTTACAAAGAActgattaatgttatttaatgatTGATGTGGTT
TATTCAAATCTATCAAATCTAAGCATGAGGTGTTCTGTTTTTTAGtagatgttattgttttaattgtgtcccacatttttta
gtattGCCTTTACATTTCGAAAGTTCTGTCCCATCATACtgctgtttaactttttttaggATATTATTGCAAAAGTT
ACGGTAGCGagagtatattattttattattgatatcgtcaggatatttttacatttaagaTGTAGATTGTCGCGGTTACGCATGC
AGCGCAGTAAGCCTGGAGTAATCCACGGTTTAATAATTCGCTTACGTCTTGGTAAAGTTATTTCTT
TGGTGCAACAATTGATCATTGTTTTAAGAGTTTTTAGAAAAACAGATACCGCATCGTCAGCACAT
GTTAATGAGTAAATGTCATCAAAGTCTGATACCTGCATCATCTTGTCCAGGGCTGCGAAGTCTGT
ACGGGTAATTGTTTTTGTACGCAGCTGATGCGTTGATCGTAAATGGGAAAGACTTATGATGACAG
TGTAGTGGTCGGTAATAGTTGTGTGTGACACTAAACATTTGACCAATAATCTAGTTTTAACCACA
ACATGATCGATGCATGATCGAGAGTGTGTCGGAATATAATGCGCAGGCATGAGTCCATGTGAGG
CGAGGAGATTGAGATAGTCTTCGGCTCTGTTATCAGTGGTAGAAGCTGATATATCTATATTGATAT
CGCcggtaatgaaaatattttggtatgaCGAAAgcgattttaaaatgttatttaaggATTCCAAGAAATGCGTGATATTTCTTA
CGCCTGGACGGCGATAGATACCCAGAATAACGGTTTggtgattaattttaattgtcaaGCAATTAGCATCTGC
TATTGCAGGTTCAGTTATGTCAAATACGTGAGAAGATTTGTAATATATTACAACGCCTTCGTTTTG
AGACATATTATCGCTAGTGTGCGCGTAATGATATCCGCTAATAAGGGGTATGTGTTGATTTTGGG
AAATCCAACATTCAGATAAAATGATGATATCCCAATCAATTTTAGATCGTGTAATCACGGTCTGT
AATCCTGTAATGTTACGATTAATGCTTCTAATATTTTGTACCATGATGTTCAACGAAGAGTTACCG
GTATATACTGATTTTTTAGAGACTTCAATATCACATGTGCTCGATTCGGCCACACTAAGTTGATCA
AGTTCATTGCTAATAGATGATTCAGAATTATGATcgaatgttaataaaaaagggTAAGCATTTCACAAACTT
GTTTATATCCAAGAATTTATGGACTTGATAGAAATTGGAGTAAATATGTAGGAACGAAATGTAtga
tacatataatttatttacgcTTTTAAATGGTAATAAGTTCAAAACATGCAGCCATATGTAATTTGTTGGGAGTT
TTGGTAAACTAAAAATGGTGTACAAGTGCTTGGGTGTGTTTAATGTATGTGTTTTAGTATTtgtaacagt
aattaaaaatgtatgtgtgtGGTGTAAACAGTGTGTGTAGCGTGTGCAAAGTAtactataatatattaaaattttgtttgaaaaaa
gaGCATgcaatatgaaaaaaattaacattatacaaaaAAAACGAACCGTTTTGACAAAAACTAGTCATACAGTGAT
CCTTAATTTATCTAGGTCTGTTGTATTGTTTATGCGAATCGGAGACGAATTTTCGTCTTTTTTGAGA
AAGACGATCCCATTTGCAGTCCAGGAAAATTTGTAGCCATGACTACTTTTGAATTGTCGCGCTAG
AAAATATATCTTCTGAGTCTTGTGTGTCAGGCTTTCCGTTACGTATAGTGGTTTCAACTGGTAGGT
ATGGTTTAAGTGagtagtatttaatttttttacagttttctttgtttttgttgaagttCTTGACACctttgataattttatcttttagcAAGACAG
TCGTTAGCTTAACGATAACCGGATTTGTACCATCTTTAGATTTTACTCTGTAAATGTCTTGTATATT
AGaatcattaatattaacattaatggTCTTACCGAGTTGCATGATTTCGTTACACAAGCTTTCTTTAGTTTCTACT
TCTTGTTTTGGTACATTGCGAATCTCTAATCCAGTTCCACATAGTTTACGCTCGGTTGCTTCTAATT
TTTCTTctaaatttttttattaaaattttatcttcCTTTTTTTCTTGCTCCAGGGATGTAATTTTTAACAAGAATTCGTCA
TATTTATTAGACAGTAATTCCATACTGGTCTTCAGCTCTCGAAATCTTAGTTCTTGATCTTTTTTAA
AGTCCGAAAAAATTTCCTTAATAACATCCTTGATGTCGAGGTCATCCTCGTCGtcgaatttctttttttccgcT
CAGTGACATTTAATGTACGAAGCTCAGGCACCGATTCACTGTGTTGTAAAGGCGAATCAGACATC
TTCTTAGCGAGTGTTAATAAGTTCACAGGCTCAACAGTCGGGGTAGATGGAGCGGGCGAGGCCG
GGGGGGTTCGTCTTAGTGGCATAATGATggtattcaatttaaattattattatataaatacagCGATTTTTATTGGTTAT
ACTGTATTACTTGGTTGCCTCTCGGAAACGTTATGCCACTATTTGATTAACTTGGGCCACAGCATA
AATAGTTACCCGCTAATCGCTGTCGCTATTGAATTGGGGCAAGCGCCGAAGATGACACCGGCGC
GTCGCACGCGCAGCGTAGCTCCGCATCTAACGCTGTCTCCGCTGTTGCTATCCCGCTTACACCAG
CACTCTGCGATCTCCACTGTATTGTGCGCGCCTAGTActtcactttattttatttatgatatttttgtcaaaaatatgaatttaaaa
ataaaaacgcagTACGGGCACACGTCCACACGCTTTCGCGGTCCGAGGGGAAGGGGGCATTAAAACGA
GACTTCGTTCTTTACTGGGGCAAGAACTGTTGGATTCGTTGATTATATTAAATACAGAATCGGATC
TAATCAAGGATGACGAAAACAACAGCTTTTACGATCGAGTCATTAACTGCTTAGCTGAAAGTTCtc

ctcttttaaaaaaaatgttgattggctagctttatataatttgttCTAAGTAAATACCTCCTtactattgtattgtattttagtatagtaataaattttt tatagtttgtttatCTCAGTATTTTTCGATAGTATCCCATCACCATGGTCTAGTCTCGGGCCCCGGGATTGT GATCAGCCACCCGGGCCCTTCCGGCCCAGTCCGACCCTG

(H).

>Hzea.12022.1 gene=Hzea.12022 "Uncharacterized LOC113506107" Log2 fold change= 4.87

aagtCGGCAGTAAGTTTAGTGCATAGGGGCTTCATTTCAACAAAACTGTACAGCCAGTGACTAGAC GAATTTAATTTCTACCTTCTATTTAAAAAGTGTTGTTGACATCGGGGATACTGTCGTCACTCCAAA CTTGACTTTGACAGGAGGAAGATATTCTCGTTGGTAACGCTATCGATCAATATCGGCCCGGTGGC GCCATCTAGCGATCCTGCACTGAACTAATACTCCTTGCCTACTGCTATGCCGGGTGTCCAGTGCGC AGCTTGCAAGCTCTTCGCATCGGCGATAGAGGGCGCCAAGTGTGGTAGCAGCAAGTGTCGGCGA TTATACCACCGAGGATGCGTTGGTTTTAAACCGGACGCAGCTGTTCCAGCATCCTGGCGCTGCcctg aatgtaaaaaaaacgtaGTCAGGGATAACAAAGCAGATACTCCTATCAGGGGTGCGGCTGGATTGGCAGG GGGGCCTTCAAGCCAAACTTGCACAGTGGCTCGTGCGTCTGGTGAGTTGGATGTCAGCTGCTCTA ACCCAACTCCCGTGGTCTGCGAACAACAACGCTCTGGCCCGTCGCCTGCTTCTTCGCCGCTACTT AGGGGCGCCTCCACAGGTGACACCGTGGCACGGGTAATCTCTCCTATCGCAGGCTCTGACTTGGG TTTGCTAATGGATGAGTTGAGGGCGATGCGTGCGGAGATACAAGAGTTCCGCAAGGAGATGGAG TTGGAGATGGCGCAGCTTGTATCTGCAATGAGCAACTGCAACGGGCGCATCGATGGCCTCGAAG CCAGAATAAGCGCATTGGAACAACAGGCTGCTACGGGTCGTTCATCGGCAGATGAGGTCGTGGA GGAGTTGAGGCGTGAGCTAAACAATAGGGATCAGGACCTATTGGCAAATGATGTGGAGATAACC AATATACCGGAGGCGCCTTCTGATCACCCTATTCACATAGCAAAAGTAATAGGCCTTAAGTTAGG GGTACAGCTGGAGGAGCGCGACATCATCAGCGCGGAGCGGGTCGGCGGCAAGCAGCTNAACGC CACTAGCTCCGCNGGTCCGGCGGTGACGCGGCCGCGCGCCGTGGTGGTGCGCCTGGCGCGCCGC GAGCTGCGCGACGAGCTGCTGGCGAGCGCGCGTGCGGCGCGGGGCGACCACCGCCGACCTC GACATGCCCGGCCCGCCACAGCGCTTCTTTATCAACGAGCGCCTCACCAAGACAAATCGCCGGT TGTTTCGGATGGCCCGAGATGCTGCTCGCCTCCACAATTGGAGATTTGTGTGGACCAAGCGCGGG CGCGTACTCGTCAGGCGGAGTCCGAGTGATTCCACTCATAGAATCCGCACTGATGAGGACATTGC ACGGATTTTTGGTCCTGGAAACAAAGCcagataa

(I).

>Hzea.27472.1 gene=Hzea.27472 "Uncharacterized LOC110379611" Log2 fold change= 4.75

ttgtaaatatctacaTAGTTATATACTTTTTTGCTTCTAGCAGTTTTACTTTTGATCTACTAGCCTTACGtaagct tttgttttcttaagtTTAAAATACGGTCGAACACTGACTTCCAACATACAGTTCTGCCTAGTTTGGACATCA GGGTCTAACGACTAATTTGTACTACCACTTCTGTACCTTAGTTTTACACAAAGTAATGTACAAGTC CATTGGTGTtcaataagaatttattattattattatttctaatgaTAATTCCACACCATATAAAGAGATACATATTGTATT TCAAGTTTTAAAACCTAGGactttaataaaactttgaacCCCATTTTTCCCTCCCTGCGGCTGTTACAACTATG TGACTTATACACAGTGAACTATGACAGTtaacagctagtttataaataaatctgctCTCAAAAAAACGACGCTGA GGAAGCATTGTAGAGCTAAAACAGCTTTAGCTAGCGAGCTCCGGCTACCCACGGCGACGGTGTC CAGGACGTCAGCTGTTATTTCTGCCAACTCCTCTTTGAACAGCCTGTTAGGATGCATGCCCCAGA AATCTTCACTTACACGTCTTCCCGAGATGTTTGTCGTCGTCGACGGCGTTCCGGTCGTCGTCGACC AATCGCCCGTCAACGTTGATGATTCGGTCGTCCATTCAGTTGTGGTCTCCGGTTCAGTGTCCTCAG GTCTGAGTGTTGTTGGTTCATTCGGTCTTCCCGGCATAGCAAACAGTCTGAAGTCCAATGTGGCTG CGGCATCAAAATGAAActttatatacaaaacattggTCAATACTCTCCCCTTTTGGACTCGCATATTAACCAT CTTGGGTTTGAGCATTTGTATCCAGGTTGGCCGCAGGTGTTTGGGTGTTTCTTTTTCACCCTTTTTTA GAGGCGGTGGGTCCGTTCGTGTTTTAAGTGGCTCATTCTTATACATCATATCAGAGTCAAAATCCA TAAGGTCTTCAGTTCCTCCCGAAAAGTCCATCTCTTCTCTAAATTCAAGATACGCTTTTCCGCGCT

GTTTGGCATTCACAGTTAACGTAACACATATGCAGGCCATGTTGTAGAGGTCGTACAATGACAGT
TTATAGGTTACCTCTCCTTTATCATTCGGCATGCGGAGGGCGAAATGCTCTCGGTACATCAGGACT
CGCCTATCTTTGCAGGCGTTCTCATATTGTTCGTGCATCTCGTCGaATCGATATATAGCAAGAGTA
TGGTTGTCTCTCTCGATAGGAGTGATAGAGTAGTGAGACAATTTGCCTTTCAAAAAGTAATGGTA
ATAGGAATGGCCGAGAATTTCTATCAACTCTTCACTACTTTCTGTTCGTCGAGTGTCCGACATGAA
CAGTTTGTGCACGGGGACGTAGTCGAACATGGTTCGGCGTCGCGTCAGCATATCTAGTGGATCCA
TCGACTGGGGGTGTGGCGTCACGAACAATTCACTACTACCCCCATTGGAGGTGACGAGCTCGATC
CAACTCAAGTACGGATACAACTCTATGTACCGGGCTGGCGCAGTACAGCCAGGACCGTAGATAC
TTAAGCCCTCCAGAGTGATAATTCCAGTATCGTTAGACACGAGCGCCATACCGTCATCCCACAGA
CAGTTTACTGCGGAGAAGGGCGCATACCCACACACGTACGAAGTAGGGATAGTGCTCTTGTCTAT
CACGCCTCTTCGTTCATTAAATCCATCGCATACTGGATTGTCCAAAGTTTGTATTTTGTGAATCACT
TTTTCCAGAGTTTCATATTCATCTGTAAAACCGGTGAGGTACACagttttggatttttaaagtGTTCTCTTGAT
AAGCACACAGGTGTCAGTGAAA

(J).

>Hzea.2506.1 gene=Hzea.2506 “Uncharacterized LOC113508874” Log2 fold change= 4.65

TTGTAACGGAACGTTACATGATTTGCAAATGCTAGTCTTTGTTTGTGCGCGGGTTAGGAACGCGC
GGAATGAACGGAACGAAGGGTTTTGATCGATGCAGCGGCGGGCGTGAGCGCAGCGCCGGATTG
ACATTCGCTCGCTGGTCGCTTCTAGACTGGTGTGTGCGTTGACCTCCAACCAAATTCCGTCGTGGC
AAGCGCCCTAGCAGACACGGccgtttaattttgaaaatcaattttattaaatcaagtGATTCAGTATATTTGTTGTGTGG
AAATCCCGAATTAGGGTAAGTTTTGAATAGCACACATATTCATATCATTCATGCAGTTATTTCCAG
CTTTGTGCTTCACTTTGTCTTTATTTGCAGcttttcatatttcaatttgtatttctGTGTACCGCTGTGCTTATTCTTGC
CGTATTGCTAGTTTTAGATGGCTTAATCTACAAGTTATAGCTGGCAATAGTTAAGAAAAGCATtagc
aaattacaaaaaatgttgcgTCATTCTAAATTCGAAGTACCTTCAAGTGTTAATAatttttccaaattaaaatttaaatattttt
tttaacttttgtaacTGCCATAACCGTTAAttctgtttaattatatttcttttcttttttttaactgttaatCAACTCAACTGTTAATTCT
GtttaattctattttcttttccttttttttgtaaactccGTTCTGTAGTACCGCCACGTCAATGCTACTACATTTAGATAGGT
AACTCATCCTCGTTGTCGTCGGTGGTACTCCAGCACGGAAGTTATTAGGCCGAGTTCTGATTAGCT
CGAACGGTGATTCCAGGTTTTTTCACCCGGCTATAAGACGCCAGCAGCAGCAGCCTCGTGTTCGG
TTTGTTACGAGCCGAGGTGTCGCCGTCGCGATAGCTTCGTGCAGCACCGCGTGCACGTCAGCACC
GTCTCCATCCCTGTGACGTCACCGTACAGCACTGCAGTACATCTCGGCCTACTTCCATCTCCGGCC
AGCGGCGTGTTTTCGCCGCCACTACAGGGGAGTACCTATCCCTGCAGAAGTGACTTCAGACAAA
GTTATACTCACGACCCACAAATCAAATCTTTGTGTGCCTCCGGCTGtggtttttttatacattatacattttttttaaaag
gtacACACccgtttttttatttctatcacCTCGTAGGTGGGAATCAAACGGGTGGAGGTTACA

Figure S2 (A-J). Nucleotide sequences in the Bt resistant strain of *Helicoverpa zea* for unknown (not found in GenBank) or uncharacterized (found in GenBank but with no functional assignment) down regulated transcripts with the greatest log2 fold change. Included left to right/top to bottom are gene ID, top BLAST result match, magnitude of log2 fold change, and nucleotide sequence 5′ to 3′. Lower case letters are tandem repeats. Additional sequence information available upon request to corresponding author.

(A).

>Hzea.14816.1 gene=Hzea.14816 “Uncharacterized LOC110381780” Log2 fold change= -7.98

ATTTAGGGTCTGTTTCACAATGTACCGATAAGTTCTGCACAAGTTCCGAATAAGCTATTTGTTACT
TATTCGTAggataaacacatttttgcgTTTCACGACTTTCGAATAGTGCTATGTGTCACATAAAGTCAGCGA

TAAGTTATGGGTCGGTTGAAGTTGCAAATAGCACAGTTATCCGACACATAAGTAGTGTGTTTGAA
GttaaaattgaataacattttctttctttcgttCGTGTTGtgttttaaacaattatatatttCGGCAATGGACATTTTTGAGACTCTTG
AAGATGAGTTTGATGAATATTTCGATAGACTCACAATACCTCGCAAGATTCCTGTGTATCGGGAA
AGAATAAACTATATGGAGACATTAGACGAAACGGACTTCAGGGCGCGCTTCAGACTCACTAAGA
AAGCTGTGATGTTTGTGTATTCAATTATTGAGAAAGCAATATCTGTCCCTACAGAAAGGTAAGTA
AGCATATACCTAACCTGCAGCCTGTATTCTTATAATGTCTCGGCTCCGTCTAGCTATTGTATGTAC
TGTAACTATACCTGATCTAACTCCAATGTTTTTTTCAGGAATTTGGCTATAAAGCCAATTAATCGT
CTACTGCTAACATTGAGGTTTTACGTGACGGGAAGCTTTCTTACAGCAGTAGGTGATTTCTCTGGT
GTAAGCAAAGCTTCTGCCAGTCGCTTTGTCTACCTGGTTTCACAAGCTATTGCACGATTGCGTGGG
TTGTTTATCAAATTTCCAACCAACACTGGAGACATCCAGCAAGAGTTTTTTAATATAGCTAAATTC
CCAAGGCTTATTGGTGCTATCGATTGTACCCATGTACCCATAAAATCTCCAggtaatatatattaaataggtact
atgaATGGTAATGTAAATAAAGGATTATTTGTCAAACTATGTACCAAATGATCAAATTGAGCTGTTT
TTATAAATgctgtttacattattttcaggtGGAAACAATGCAGAAGAATGGAGAAACAGGAaatcacaattttcttttaatg
tgCAAACTGTAGTGAGTGCAAAGTTAAGGATCTTGGACATAGTGGCTAGATGGCCAGGAGCAGCT
CAtgatcaaacaatatttaataattcttttataaaGCAACGACTTCAAAATGGAGAATTTAGAAACTGTATAATTGTT
GGTGATAGAGGATATGAAAATACTTCATACCTACTTACTCCACTACAAACTCCCATGACTCCAGC
AGAACATCTGTATAATGAATACCAAATAAGAAGCAGAAATGTAGTAGAAAGGACCTATGGGGTT
TGGAAAAACAGATTCcctattttatcaaaaaaaatttttGCTATCTGTATCTCGTGTACAGGCTGTAATAGTGGCA
TGTGCAGTGTTGCACAATATTGCAATAGATATGCGAGATGACCATTTTGAAGCCATCTACCCCGT
GGGTGAGCCAGATGAACACGATGTTGTGAATCAAAGTAGTCTAGGAAATTTGGGGGATGCAACA
GTGcgaagtaatttaattaatgattattttgctTCATTACTATAATAATGACAGTCATTTATAGGCATTTTGCATTCC
ATTAATATAGTAGGTTATTAAATGTATtctgtgttaaataaaaaacgatttatttttcaaatttaaaaagtattttttttccaacgCCCA
CATTTCTCTTTTGTGCTGCTCCCTTTCTTCCTCTTCGAAGGCCTAttgctttcttttatttaacaattctTCCTCCAGA
ATCTTCTTCTGTAAATCTGCAATCTCTAAATTTGAATCAGCTACTCTTTCGAATGGCCTTTTCATCT
TCTCAGCAGATAAGGCAGGATGTTTTTTGGAGCGCAGAGATTTTGGCTTCCAATTAGTCCACTTCT
CATCATAGGCGACTTTTTTGGGTGTAGTATATTgacttaTCTTTCTTCTGGAATGCCCTTCCTCATCAG
AGTCGTCATTTTGTGTAGACTGACCTTCCGTTATGTGATGCTCTATATTGGTATCCACTGATTGGAC
ACAGTTATTGTCTGCAATAATCTccattatttcaatgttttgtgttaaaatagTATCTTGGTTGGATGTGGAAGGTGT
TGGCGGAGGACATACTGAGTCTATTTGTATAGGATctgcaacataaataaaattaaataagaaagagAAGGAGCAT
GACAATTGTATTATAATTcacttacatacaaaatactgaTTGGCTCGTCATATTAGTAGTACAGaactaataacaatttt
aaacttatGATCTTACCAACATGATCAGAATTAAATTCACTTTCAATTCCATCTACAGACAACAGTATC
ATGTCTTTAACTTTTTCCTCTAGTGGCGTCAGAGGAGGAGCAGCACTAGGTCCACCTCCCGTGCG
ATACGTTTCGGCACGTATTAGAGCAGACTTTTTCCTggtatttctttttataccTTCGTATTTCGCTTTCAAAGT
TTTGGCACTGCGATAATTAGTACCGCTTTTGCTATTAAATAGTGTCTCCAATGTCTTCCAACACTGT
TCTTTTTCGTTCCATGTGACTGCATcacttttcttattttctaaaatgCTTTTATGTTGAGCAACGAGTGAAGTTA
GAAGCTCAACTTCTTCCCGGCTGAAGTTAACGCTTCGTTCACGTTTCGATgtcatttttcaatacatagAAAT
GACTGTGCATAAATGTATTCAAGAGGTACACTCAAATCGCAAGAATAGCAAAAAAA

(B).

>Hzea.32212.1 gene=Hzea.32212 “Uncharacterized LOC110375279” Log2 fold change= -5.76

gCATGTTGTTTGCTGGATTTAtttatgcttttaaataaataaagttgagcCTTAAGTTTATTGagattataacaaaaaacaaaaac
atcgtTATCGACTAgacataaaactattaaatcgCTGTGTTAGCATGGTTTGTTCAAGACTATGTATGTTAACGG
TGAGTGCTCTTCGTTTATGGATGGTACTCCATAAGATCTCCTCATAGCTGCTTGAATACACCTGAT
TATGTCATGATACGGCACGACTTCTTCTTGTGGCGGCTTCAGTAACTCAAATGTTTCCTTATTCTTA

CCATTTTCCCCGGTTTTTTCTTCAGTAGCTTTTTTCTTAGGTTTTTCTTTCAGTTCTTCTTTCGATTCTT
TAGATACAGACTCCTCTGTAGATTCATTGGAGGCAGGTTTTGAAGCGCTGTCTTTTTCTTCAGATTT
TGGTCCAGATTCCTTGGAAGATGAATTTTCCTCTTTAGACTTTTCGCTAGATTCTTCTTTGGAGGTT
TTTGGTTCTAACTGtagattttcttttgatttttccCCAGATACATCAGTAGATGGTTCTTCGATAGATTCGGGTT
TTGTTTCGGGCACTTCCTTTGACTTATTTTCAGTTTCTTCCCCAGATCCTTCTTTGGACTGTGTTTGTT
CTTTTGAATTAACTTCCGCACTTTTTTCATTAGAGTTGGCTTTTGATTCTTCCTCTGACACGGCTTGG
GTTACAGGttcagtatttatttctttactattttCAGCAGATACCTCTTTAGATAGTTCTTTATGTAAGTTTTCATGA
ATTTTCAATAGCTGCTCAGTAATCGCTTCCGAGTTAACGGTTGAACCTTGTTTGATGGATTCGCTTT
TGATTTCATGACAGTCAAAATTCAATGGCGGAGATGATTCGATTTTCACAACTggttcaattttctttttgacT
TCCACGGTGGCAGGTAAGAAATTCTTAACTATCGCATTGAacccaGAAATGTCGTCAGCTACATACT
GCACTTCCCTAACATAGCCGTCGGGCTGAATAAAAGAGTATTCTCCTCTTACAACTGAGCCATCG
CTAAGTTCACGTTGTGCCTTGTGGTCTCCAGTACTCTCATCATACACAGCATAGGAATATTCATAT
CCGTTGGTAGCCTCTTTAGGCGGAGTTACTAACACTGGTACATTTGGTACCGGAGCACTGTAAGC
TATTACATTTGGACTAGGGTTCACGGGAGCCACCACAGAAGAAATAGCTTGGGCAGGAAAAACA
CAAGGTGACCCACAAGGTGCACCATACTCAGTAATTGCAGGGAGTGAAGTGATTTGAGATATTC
TCGGGTCTGCTAATGACGTGTAAGGGGCTCCATATCGATAATATCCCCTAGGGGCAATGTCACGC
TCTACATAGACATTGGAATACGAAGCACCATTAACTCCGCTCAATATACCGATGCTGAATATTAT
TGTCACCACGATAGCtaaaacaaTCATCTTTCCTGGTGATCGATGGAAGACTACTGATATTTTTGAGA
ATGAAGCTGCGCTTATAAGAGCTTCAttgtttgtc

(C).

>Hzea.31665.1 gene=Hzea.31665 "Unknown Gene" Log2 fold change= -5.56

ggGTCGATTTCAAATTTGGGGCCAAAAgcatataatttcatttatttctcatGATGTAGAATTTATcgtaactaaaattatttt
aaatcatgtagTCTTATGAGACGTCCAAACGCCAGTTAAAACGAATCTTTTatgattgtaaattaaaaagacATATC
TAAAACTAGTACCTATTTCTCATTAGTGTTAATGCTCCAAAgtcttatttttttctaaatctcTCTAAAAAACGAA
ATAACGCGTCGaggaaatttaaattttactgtGGTATTTTTCTGTATACACTTTAAAGATGaataattagatattaaaaCG
AATGCtgggtataaaaaaatatgtttttttagctttataaaaCTGTTGGTTTTTAAAGTATCATTTGTGTGACCGTATTA
AAATGCATCAGTAAACAGAATTCGTCCCTGATTTACCCTGCCCTTTACCCCGCGCGCGTCCCGTGT
GCTCAGTCGCGACCGAACGCGCGTGCTCGTCAGTTGTGTGTGGCGTTATTTTAAGAGAAATTCCA
AATTTCAGGTAAGATTTTGACTTTGGTAACAAATTTATATCATTAGTGTTTTGTCACATATTGTGCA
aagttaaatgtgtttttttgtttttgatttggCTGTTTAACAAACCATTGAGAGCATATTTTGTTTCCAATGTACGGTAT
ATTCTCTAGAACGttctatttacgaaataaattcGAATTACTTCCAAAAATCTCATTCGTGTCACTCTCATTTGT
GTCGATTCACGAGTCACACGAATGATATGGAAAGTCAcactaatgattttttttttgtttacttttagattaagtattaaataat
ggcgaaaccaaaaaaacaaaaaagtgagGAAGAATTAAAGAGGCGTAGAATAGCTAGAagacaaaaatatcaaactatTA
AAAACAATCCTGAGTTATATGCCGCTgagcaagaaaaaaaaagacaatattaCTTAAAAGCGAAggagaataaaaaa
attaaaagtataaacGAAATGAGTACACGCGAGCAAAGAATTCAAAGACGAAAATGGAAAGAGAGtgcca
aaaagttttatagaaaGAAAGTAAGCCAAAGAATTATAGAGAACGTTGCAGTAACTGAAGAAAGTTgtgata
ttgaaaatgttaacatGAATACAGATcctttaaatatagaaaaccaagaaataaaatccaaaataagTATGCGtgaacaaaaactaataaaa
aaaatcaaaacaatgaaaatgcaGCACGAAAAAGAGAAAcgaaattatatattattattaaataggtacaggttcaggttaaattatttaaaa
agaaaaatatctacacTAACAGGTGAAATAAGTAATGAatcagaaaatacaaaaaatgaaGACAAGAAACAATTATT
TGATAGACTTCTAAGTGCTGAAATAACACAGAAATTTGCAAAAACTGCAAACATTAAAGACAAA
CGCGTTATCACTAAGTTAATTGATAAAGAgaagtttaaaaaatttaaGGTTGCTACTGACCTTCGGAAAGTT
CATCTTCGAGAACGATTTAAGAAGAATGCACGTAATACCATCAACATTCAAAAATTAGTAGAAA
AGTTTTACGAAGACGACATTAACAGTCGCATTACTCCAGGCAAGAAACAATATAAGAAGAAAC
ATGGAATCGTTAAACAAGTCAGATATCTTTTAGATTCTATAAAAAACTTACACAAGAAATTTTTG
GCTGAAAatactaacattaaaattagttaCACGACTTTTACAAGACTCAGACCATTTTGGGTCTCTCTTCCGAC
TGACGACCGTGATACCTGCGCCTGCTTGTTGCACACAAATTTTAACCTATTAATAGCGGCtcttaataa

gaataatattctTCATGTAAAAGATAGTCAGAATTTGATAAAGGAAATCTGTTGTGATAAATATAGGCT
AGATTGTCTTAATCGCAAATGCGCttcatgtaaaaataaaacagtacctTATAAAGAATTCCGAAATGATAAAG
AAATACAGTACATGGTATGGGAAAGAGACACCAAAAAAGTAGAcacgaaagaaataaaaataatcaagaagA
AAACAAGATCCTCGTAACCTGATAATTGAACTGGAAAATATGCTGCCAAAATTTTTGTCCCACAC
GAGTAACATTGTCAACCAGTACGCTactattaaatgtttaaaagaatCTCTAACTACTAATGAAGCCTTGATTC
ATATGGATTTTTCGgaaaattattcttataaatattcgGAGGAAGTACAAAGCATGCATTTTGGTGGCAGCCGTG
GTCAAGTCTCACTTCATACAGCagtggtttatttaaaaaatgattccAATGCTACTCAAGCCTATTCTGTTTGCAC
AGCCAGTGAATGTACCCGTCATGATGCAGCGGCAGTGTGGGCtcatctaaattatttaattaaatttgtgtTTAACA
GATCATCACAAATTAATCGCATTCACATTTTAACAGATAGTCCCACAAGCCagtacagaaataaatacatattt
tatatattaactcAATTAAGGAATGACTTCCCAGAACTAAATCTTGTCACGTGGAATTACCAAGAAGCTG
GGCACGGCAAGGGTGCTCCTGATGGCATTGGAGCAGTGATCAAAAGGACTGCCGATTATGAATT
AAAGTGTCATCAGGATGTCGGAGACTTTGATGCCTTTGTTAACATTATtcgaagaaatataaaaaacgTAGA
AATGGGTATTATTCAAGAGCACGAAATTAAATTTAAGGAATTAATGTTGCCTAAAGATATTGCCA
CTTTCAAGGGGACTATGACAGTTCATcaggtatattttatacaacaaataaacattaaaaaatatactgatgTTACCATAtaca
atgtaataataatacatatatttttctttcaggtCGTTTGGGCAGCCGATTCTGCTTATCTAGAATTTAGAAAACAGAG
CTGTTTCGAATGTATAAGTGTCGCTTGTAAACATGGCAAACATATAAGATTTTTCAAGATATATA
ATACTCCAATTGGATCTACAATGGAAAATGTAAAGGTCATCCCGGAAAAGAAAAGAATTAAGGT
ACTTAGTGATGTTACCATTCGCTATGCTAATACCGATGATCCAATGTTACATGACAGCACGCCAT
CAACTTCAAGTCTCCGCAGTGTTCGACACTCCTGGgccaataaatgttttataaataattatacaaaagcGACAGATC
ATGAAAAAGAATTCCttgattttataaagaaatgtgaagatgatgatgacgaccccttatcagattaattaattaagaaggTATTAATA
ATATGGATATTGTTATTGCACACGCAATGAAAAAGGATTAGCGTTAACGAATCTTGAAGACTAA
AATCAGAAATGTATACCATTGATTAGCTTAGTTgaataagttattgttttggaattaagtttaaaataattaatatctgtAGTA
CGGTGACCAGCACATCCACCTACCCCAATATGTCAAAGTTTCCTAATGGAATTTGACGCTTTTTAC
ACTGttgcaataaattgaaaatcttttaaagaAATTTGATAGTAGGTTGAAGCGCTGGCTACTGCAATAATGTTAC
TACCCTACCTactattaataatgttaatgacaaatattttatgttttgttgattatttattaattttcattagtGTAACCAGAGATCTCATT
AGTAAATCACAAAATATCATTAGAGAAATTATGTTGCTCATTCGTGTCACtcaaaaatgtgtattttcCCTGT
TATTGGAAGAAATTAAAGGTTTGTTTGATTAgcatatgttttttttaactaatGAATGCACCTAgatatataaacattttttt
ttaatatttttcaaatatcatGCAATTCACACTTTTTTGGCCCCAAATTTGAAATCGACCC

(D).

>Hzea.25802.1 gene=Hzea.25802 “Uncharacterized LOC110384548” Log2 fold change= -5.56

TAGCGTTAATCGACGAACACAGACGTGCTCATCAAGCAACTTAAAAATAGcgtaattaacaattttttattgtg
acctAGTTTTAGAGTCATAGCgtttttgttgtgaaattatacagtatttattacaaatatggACAAAAAGTGATCTACAATTA
TGGATAGAAGCCCCTCCCACCGTGGCGGACTGCCACATTGCTCATCGCCACCAGCAGCAACACC
CAGAAGTCACGCACAGGATGAAACAATTGAGGTTTTTTCTACTCCTGAAATTCAATCCTGGATGT
CTAGTATTGAGCAGTGCCTGAATGAGGTTTCGATCACGGTTTCGGAAGGCAAGATGAATTCCGAA
CAAAAACTAAGAGTCACTAGCCTTTGTCGCAAAGTCGGATACGGAGTCTCTCAAATGGCTGTGC
AATATCAGTCATTGAAACATAAAGCCCTTCAGAACTACGCCACCCTGCAAACGTTGAAGGAAAA
TCAAGACCTCGCTAGTAGTTTGACTGAGATCAAGCAGGAACTACAAGATACTCTCATCAAACAT
AAGCCGGAAAGCACATCCTTCGCAGACATGGTGAAGGGTACAAAAAGCCTTATTATGCCTCATG
CAACCAGCTCTGTCGCTATATATCCTAGTGACCATACTAAGACCAGTGAAGAGACCAAAAATTT
AGTCCAAAAAATTGTATGCCCTGAGGAAATGAAGCTAAAGGTACGTGGACTTTGGAAAACAAGA
AACGGAGGTGTTATCATAAGCACAGAGACTAAGGATGACATGCTAAAAGTGAAAGAGACTGTC
GAGCGTTCCACATCTGCACTTACTGTTGATGAACCTCAGAAGCGGAAGCCTCGCATTATAATTAT
TGGGGTACCCACTGATATGGCAgagaaagaagttttcaaaagtatttatgACCAAAATATCGTAGACAAACTTCC
AACATTGACCAAGGATACCTTTTTGACCTCAATTAAACTAAGCCACAAGTCAGGAAAAAGGGAT
GCTGACAGCTGCAACTATATTGTTGAAGTTCCAGCCAGCATCCGGAAAGTCCTAGTAAATCAGA

ACAGGCTTTACATAAACTGGACGTCGTGCCCGGTACATGATTTCACCCTTGTGACTCGGTGCTTCA
AGTGTCAGCAGTATGGTCATGCATCCAAGACATGCAAATCTGCTACCTCTACTTGTGGCCACTGT
GGAGGAGAGGGACACGCGACTCAAGAGTGCTCTTCAAAGGAAGAACCGCCGAAATGTGCAACC
TGTAAGCGCTTTAAAAAACCCTACAATCACAAAACGGGAGATTCCGAATGCCCTGCTAGAAAAG
CCGCTGAATATAGGTACATCAACTCCGTAGACTATAAAGGCGTCTGATAGCGTCAAACTCGAGA
TAATATTTAGCTGTAATCATTTCTAcacattatataatttcatattgtaCTTAGGGATAGTGgattactgattatttttaggCC
CGCATATTTACACtcaaatattgtatgtaatattgtaggtacgcaatatggatattttaattagtatgtAAGCATTATAACAAACAT
GTGTAATAACTCTGTATTTGTCACATTGACCTAGTTTTTAGTAACTAGcagcaatttattgttaattagctataaatat
tataataagcactttTACGCCCGAGACACAGGTTTTTACCTAGTTCGGGCAAATTatacatcagcacttttagaattagca
aaatattgtaaaatgatGTTCAAAgagaagtaataaacaatttat

(E).

>Hzea.6443.1 gene=Hzea.6443 “Uncharacterized LOC110383326” Log2 fold change= -5.02

tttggAAACAACATGTTTAATCTTCGAAAATATGCCAAACTGAGACATTATAATCTGCAGCAAAAA
AGTCTGATTTTGATGGATCAACCCTCGAAAACACAAAGAGCTTGTCTGGGGGTTTCGATGAAATA
AACATGCATGGTGACCACGCCGGGGTTTTCTGCTAACTCGTAGAAACATTCGGGACCTTTTGGTA
CGATgacCTTAACAGCTGCTACTCCAGGACTTTcgaaataattgaaattgtCAGGTCTCACGGCGTATTTGCC
GATAAAATTGAAGCCAATGCCCATCACTACTCCGTTGTAGGAAAGTGGGAACCAGGGGAACAGC
CTTCCTTCAGCGCACTCCAAGCTTGTGGTCATGTTGTAGTAGAAATGGTGACCCATATATATCATG
CAGGCTTGCTCTGTGAACCCACTGACGCTCGTGTCGGCAAGTACGGTAGGGTCGGAGGAAATCG
TGTACAATTCTCCGTTGAAACCAGTCACTACCATGGAACCACCCTGGATCAACGTCTCAGTATTT
CTTGAAGAGTTGCGAGCTGTTGCGTCAGTCGCAAGATAAtCAGCAGTGGAAAAGTATGCTCTGAT
GGTCCAATAATCTCTAGATGTGCCATTTGACAATGTAGTGGTCCAGCTTGTGAATCCTTGATTTTC
GAAATCGTATACCGAATCGCTTATTTGTTCTTGGTCCAGACCAATTTGGAAAGCAGCAATCTGAT
AGTTGTCATCGTAGTATAAGTTGAGGGTGTTGTCATCAGCGGGGCCATAAAGGACCAGGTTCTCA
TAGCCATTTGGGAGGGTAGGGCCGGTCTTCTGCACCCAGTTTTTGGCAATCGCTTCCCATATAGTA
AGTGGCATCTCGACGAAAGATGAGGAACCGAAGCCGAACGGATTCAAGTCAAACCTCACCCTC
ATATTGGCTGTGAAAGCACCGAAAGCCGGTGCAGCCACGAGGCATAGTAGAGCGATTGTCCGGA
TCATGGTGGAAACTGGAAACTGTGT

(F).

>Hzea.17664.1 gene=Hzea.17664 “Uncharacterized LOC110376085” Log2 fold change= -4.97

tttgttatAGTTGGTGCTATACCTTTTCAGCGTCCTCAATAGCAGTGTCAACTTCTTCAGGTCTAAACTC
TACACTGCCATCTGAACCAGTATCTTCCAAACATTCTGGACACGGAGTAGATTCACAAGGATTTT
CGGTTGTCGTCGGGAATGCAGGCATCAGCTCAGTTGGTGGCTcCTCAAGCCTATACAAATAGGAT
AATCGACACGGATCCCACGTGGTGCAAAGAGTAGCGTTGTGCATCATATCCTTCCGTGGCTCGTC
ACCGAAATAGTCGAATGTGATTACTATATTATTGTCTATCGCGCATTCTTCTAGAAATTGTGTCAC
GCTAATtttagTTTTGTTTCCGCAATCTAAAGTTCCGTTCTGGAAATAATTGTTTTCTCTCTCACCATT
ACCGAGACAGAACATCCAGTCTTCACCGAATGAATACTCTATGGGAGCTACCTCGGCTTCGATGC
AAGGGTCCAAGGCAACTGGAATAGATTCTTGTCCGAAGGTACATTTACGTTCGTGTAAATTAGTC
TGCACTCGGTAAGCGTCAAGGTCTGGCTCTGAACCTGGTGGACATTTGAAGTAGTTTCCATTCTGG
ATAATGTAAATAGATTTCTTGTGATTACTGACTACCGTCCTGACGGTGAGACCGTTACCGCCACTC
TTAATGTTTTCCATGAGCACTTCTTGAGCCGCGCGTTGTTCACGAGACCATTTCGCAGCCTCTTCT
GCCAAGATCTGCCGACTTACCTTTCCTGGGGATTGAgaagaCTGTGGTGGATTTTCCATAATAACGT
GTGGTCTTCCTAGGGTGTGCTGCCAAATAAGTTGCGCAGCAACTTTAAACTTGGAGCTGTGGTGA
AGAGGTTGAATTTGTAGAATACCATACCACGCTAGCTCTCTGTACACGCTGTCCGGTAAGTAGCC

CGTAGGCATGAAGTCCATGTGCcactgcaataaaatatataatgtaatgaaaacaagaatattaggtatgtattgttatttattttggaga
AATATATATTCTTACTTCCTCTTCTTGCGTATCTATGGCTGTTAACTtatctgtaaaaataatatggataTGAATA
TACTCTCAAGTTcttcataaaatgtttcttcAGAACCAGTTATCAACTTGCCTTCATCGTCATCAGAATACGCT
ACAGctagctgtaaataaaattaaatataataaatgaatagaTCCGAACTTaatatttgaattatatttgcGTGCCAACCTGTAGGA
CTAGTACAACAGCAAACGTTATGTAGTTCGCCATTCTTGAGAAAAGTGAAACTTTAACTAGTTGC
TACCTTTCCGACggccaattaattaattttaaagaaaatgtaataaaaaataattgttacttcgtttacatttatttatgtgaatattGATAAGG
ATTTATATGAGTTAACAATACTGTGGCAATACATTGCGTAAATCGAAACGTTCAATCTGCATTCTG
TGtacacagataataaaaaagCAGCTGTCAGTTTCAGTTTCGCA

(G).

>Hzea.32214.1 gene=Hzea.32214 “Uncharacterized LOC110375279” Log2 fold change= -4.91

GTCAGATTCACACTGTATTTAAGAATTGCATATCACAGTCATtacgtacaaaataataaataggcacAATACAA
TCAGTCAATGTTGATTATTCAGTAGTGGTGGTAGTCGTGTCCGTGGACGGGGGCGGGGTGGACCG
ACGGGGCGGAGTTGTGCACCACAGCgttgaaaCCATGGTGGTCGTCAGCGGTGTACTCGACCTTGCG
CACGGAGCCATCAGGCTGCACCAGAGAGTAGCTGCCGTGCACGGCGTCTCCGTCGCGGCTCTCG
TGCTGGGACTTGTGGTCACCGGTGTGAGGGTCGGCCACGGAGTAAGAGTAGTCGTATTTGGGGTG
AGCGTAGGTGTCGTGTCCGTCGTAGTGGGCTACGGGGGCGGCGTAGTGCGCTACGGGAGCGGCG
GCGTAGTGAGCGACGGGAGCAGCGGCGTAGTGAGCTACGGGAGCAGCTGCGTAGTGAAGGGGA
GCAGCATGGCTCTCGTCGTGACGAATGATGCTCTGAGAAGACACAGCGTGGCCATGTCCCAAGA
GACCAGCGTTGGCAGCAGCCAAGAGGGCGCCGAAGGCTACGATTTTGCTGAACATTTTTAGTGTT
TAGATTGTAAGAATCAAAAGTTGATTAATGTCAATGGTCCTGCTGTATGGTTTTATAGAAcgcgggcg
gcgcgtgcgccGGCGCGGTGGGGCGGAGAAATGCGCGCGCACCTTGACTCTATAACTTTGACATGTAT
CAAGGTCGCAGTTCCTACAATACGAGACTAATACGTATGCATTGATTTGTGTCCGGATCTAC

(H).

>Hzea.10608.1 gene=Hzea.10608 “Uncharacterized LOC110373805” Log2 fold change= -4.60

ataataagcccttTTATTCTGTACTGCGTGCACGTAGATAGTATTATGGTGTAgaagtttctttataaaatgtCCCGCA
GTTTAGTGTGCCTGttggcataggcctcctctaaCATTTTCCACTGTGTTCTGTCTCGAGCTACCCTTCTCCAGT
GGTGTCCAGCCGTGAATTTGAGGTCATCTTCCCATCTCATCTGTTGGCGTCCTCTATTTCTCTTTCC
GTCCCTGGGATACCAGTCTGAGACGATTGTGCTCCATTTGTTGTGTGGATCGCGAATCATGTGGCC
GGTCCACCTCCACTTCTGTTGGTCTATTCGAGTAAGTATGTCTACTACATTGGTTCTTTCTCGTATC
ACGCTACTTCTTACTCTGTCCTGTATCCTGTTCCCTGTCATACTCCTCTCCATTGCTCTTTGGCAGC
GTTGTAGTTTTTGCCTGTGGAGCTTGGTGAGTGACCAGGTTTCACACCCATAGGTGATGACTGGA
AGGATGCATGTGTCAAAGACTTTTCTTTTGGTTGACATACTTATCTCCTTGGATTTAACAATCTCTT
TCAGTGCCCAGAACTTTTTCCATCCGTTTGCGACTCTTCTGTTGATTTCCTTGGTTGTTTGGTCCTTA
TGGGAGATTATCTGACCTAAGTAGATGTATTCTTGTACATATTCTAGTGGTTGGTCGTcgatttttatttcta
ttggtTTTGAATTTGTGATgagtttggttttgtttttgttcattgTTAAGCCTACTTTGACGAAGTTCAGTATTAAGAG
GGGGGCAAGACAAGGGGATCCCCTATCACCGAAATTGTTCTCGGCTGTTCTCGAAGGTATCTTCC
GTAAATTAGACTGGAACGGATTCGGGCTCAACATTCTAGGGACAAAATTGAACCACCTAAGATT
TGCGGACGACATAGTCCTGTTCGAAGAAAACCCTGCACACTTAGAAGAAATGATAAATTCACTT
AATGAGGAAagtgt

(I).

>Hzea.26537.1 gene=Hzea.26537 “Uncharacterized LOC110373805” Log2 fold change= -4.41

TCGAAGTCACTTTAAACTACTAACTAGTagagatatttttgtatgtttccAGGTTTACAGGTATCAATGC

TTGATATCTCTAATGCTATCCAGCCAAACAAAGGATCAAGTAACATTTGCAGCCATGGAACGATT
GAGAGCCAGAGGGTTGACGATAGACAATGTTTTGGCTATGAGTGATGATGAACTGGTGTAACATT
AATTGTTACATGTCttaattctttttaataaaatgttgagtaaaataaaattaacaaaactatttacaaaactaaaactatttacaaaaactaaacta
tttacAGTAGCAAACTAAACGCATCAAAATTTCTGGCCAGCTACATTTTATCACCACGCACGTATTT
AAACGAGCGACATATTAGGCACCCGGACCGCACCAGCGACTCCTCGCTTCCACGTCGCCGCTGT
CACGGCTGCTGCCAGTGCGAATCTGGATATCGAGGCGAACGCACGCCGGTTTTTTGAACACTAAC
TTTAAGTTATTATAACCGCCATAGCGATTAAACTTTTTAAGGGCGTCATTACCGTTCCCTCGACTA
TATATCTCGACATATAATAATACTCAGCATATTCAGTGTGTGTGATTTAAACCCCGATTGGGTAAG
tgctatacaattttaaattagccAGAAGCTAAGCAAACCATAACGGTTTCTTTCGCTTTGTTTTAGCTGGGTAAT
CTCTTTATTTGTGTTATCGCTTAACTTTATTTCTATTGCAAGcttattatgtgtttttgcaTAGTAGTTTACCCCA
CACTTtcttaataatgtaatttttgccTACTTGCGTTTTAGTTAccgttaaaattttcaaattttgaatTGGCTAAGTAACCGACT
TATTGTTATCTCCGTATCTGATAGTGAACATCCACAGCAACACATCTACACTTTTAGCGCTTCCCT
ATTCAGCGTTGCTGTGGGTGCTCACCGTCTACGGCCAGTGTGTAATGCCTTCGCTTATTGCGCATT
ACAGGGTTTCACGTACACCTTAAGTCTCCTGCTTCCCCGCGGTGGACTGTAGCGGGTTGACCTTCG
AACCCTACACCTACACCTGCACCTGGACCTGATCTCGGCGCGCGGCCACTTGTTCCTGCAGCGAC
CAGTGACGTCAGGGAAGTGCTAGGCTAGTGAGGAGGTTAATCACTGTAAACTTGTATGTATGGG
CTCTAACCCATTGTTATTTCTGCCGACCGAGCTATACTGTGTTTGTGTGCGGTAAATAAATTGTCC
ATCTTgctacctaccttttttttatttaataaccccTGGGAGGGATAAAGTAGCGTAGCAGAGGATAAGACCGGGAC
ACTGGCGCCCTTAAACGTGGTAGAAATACAGTTGATTACTCCTCCTCCTTTGGTTGTGGGTTGTGT
ATGTTAGTGGTTGTCTGTGTAaacgtcttttatttttggtttttcCGTTACTAATGTGTACGTCACAAGCTTTCAA
TTTCTTTGAGCAGTTAATGTTggtcataatttattaatctaccctacattaatttttattaatttaaagtcGTTTAGTTGCATAGTTT
GTGCAAATATTTCTTGCTAATGTTTTTGTCTCGGGCACCTAAAACGACGCtttcttaaaattgtttttttttttcatta
taattttttattgtgttcacatttaaataaatatcatactAGTATTGTATTATTAGTTAATTAAGGTACAGTACACATtagtagc
atttttttttatttcttctgctTCTTtcttttatgattttgttttttttttaatttgattatttttttttcgttaaaaaaaaaaccagacgttcttaaattttaattgtaacag
AACGGAGAACGTgttgttttttctcttttaattattgttttttttttctcttttaagctttgttttttttttagttttgtcattgtttttttctttaaatttttttgtttt
gtgttgtgaGTAGTAGCGCATGCGTCATTTATCCTTCTGTTTTTAATTGATCTTCAATATGCTTCCTATTA
AGTTCCTGTCTCTTCAAAAATCTGAGTTGGAATATGAAGTTCGTATTAGAGGCGCGACTCCTGCA
CCTTCTGTTGAGGAGTTacgaaaacaaattataaaattatccgCTGATTTACCTTCAGAATATATATTAGAGTCA
CCTCTTGACGCAAGACAGGACCTTAAGGGTTGTTTAGAAGTTTTAacgaaaattcaattaaatcttGACGCTA
GCGATCCTAGCGTTGCTTCACTATTGCGGACACAGAATATGTTGAATCATTTATACAATCGTGTCG
AGAGGATAACGCGTAGTGACGAC

(J).

>Hzea.30333.1 gene=Hzea.30333 “Uncharacterized LOC110380598” Log2 fold change= -4.38

gtcagataggccgtcgccccttgtaaaactctggtaatcagctgcatccaattagactAGAAAATCGACCCCAACATGGGGAACGa
gggagatgatgatgggtaatggtattataaaaaaaagaacgtaaaatgaaatattaaacttctattttcataatataggGTGACAGGTGATGA
GGCATCGGCTTAGAGGTCAGTGACGAGATAAACATATCtgcatattaattaagtaataagactgccacttttacttttaagt
ctttttgtgacaaataaaactttatatgtTTCCCAATGTTTTGATTTTCGGAACAAATCATACTGAGTCTTCTGTACA
GCATTCATATTGTTCCGATCACCTTTTTCTATAATTGAAAGATCTACATTAGATTTAAATAGTTCGT
AGAGAGCCTCAATTGCTTGTCTGTCGTCAGCAATCTCTTTAGCTTTTGATAAATACGCTTCTACCA
ACTCGTGCGAATCGAAGAAACTACCTAACATCTCGAGAAACGGATTTCCGTGATCCAGAACATC
GCCCAGGTCAATCCCCTTTAGCAATTGTGTCGATTTAGTCATGATTCTCTTTTCTTTGTTGCAGTTA
GATAAAACGTTGCCAATATCGGCCATCAGCATTTCCAGCTCCATACGAAATTGTGGATCCTGCTT
ACATTTCTTAATGAGTTGGTCATTAGGCTTATTTGGATATTCTAATAAATCGTGGAGTGACTTTAG
TctactagtaaaaaaaaaataacagtgcaGTGTTctctattgtttgtttttttgtgacCTTtgaagttatattataaattgctTGTATACCACTAA
ATGACTGATTTTCTAAATTCAAGCCATGTTCATAAAGTAATTCCATTTCATGTATTATCTCACGTG
CATTAATTAAAGTTGCATTATCTGGCTTTTCATTCAACAGTTCCAAGCTAGGACGCATTGACGCAA

ATAGTTCACAGTTGCTGATACAAAACGTCAGCATATCTTTCATAGACACCCAATGAATTGAATAA
TCATTTATTCTGTTCAGAAGTTTCAAAATATCGTTTTTACTCGAATTATTTCCATTTTCTATTATCTG
CCTttgattattaatattattcttatcAGGATAATCCGTTAGTGTGACTTTAAATCTATCTTTCTTTAACTGTTCTTT
CAGCAGATTcacattatttgtattttcctCTATGAGTTCTATCGTCTTTCTTATTTTGGCTATATTGCTGGGCAAC
ATATTAACTAGCTTACAAGTGAGagtttctttaatttcttgTACTTTTTGGGACATGTCATCTTTATTTTCCAG
AaggaatattatttctttaagaaGCTGAATCGTAAGACCTGCGGGTTTCCAAACTTGTTggataaagtattttttttcgcaaaa
caagcaatttttattggttttatattgGGTTTGAATTTCTGATAGTGTTTTCGGGAGGCGGGGCCATTCTTTAGTTC
GTTTCTCTAAAAGGCTTAAGCCCTTTTCGACTAAGGATATATCAagtatacatttattaatttcatgaaCCGAAAT
AAAGGTTTTCTTCATTTCTTTCAAGTCTTTGACAATATTAAGGAACATTTGCATATCCTGGTACTCA
GCGCTGGTCCTCACGGTCAGGTCTTCATGGTCGGAGAGGAAATCCCTGATGTTATTCATCTGATTG
GTAGTCTCCACCGATAGTGATACTTGTAGAAGAGCTTCTGTAGATTTTGATACATGATAAGACTC
GTCCGTACTTTTTGTCATTTCGCCGATTACTTGAAGGGCTCTCTCAATCATTAACACACTAGGGTC
ATTTGTTCTTTCGATTGCTTCtacgtaatttattatttttttgcaatgaatAATGATCATTCATATACATAGTTGACCTT
AGGTAAATCTTACCTTTACATTTACGTTTTCGgacttcttttctttttttctttttctgtttctttatttttttttagaagctgctttttttttcttttt
agaagACTTTGGTGCTCCCGACGAGTCTGATGTAGCCGAGCTTTGTGTCACTGGGCTGGTATAGTTT
TTATAGTatgtaagtatttcttttagtttgtccaaattaattttgaagttttccctttctaaatataaaaaaggccCTCTTTTCACTTTTTCGAA
ATCAATTACAATTTGTAGGTTTTTTCGTACGCGACCATCGTATCGACCCAAGTGATTTTTCTCCGTG
ATGATTTCTCTGTCACTTGATAGATACAGCCTCCAGTCTTCGTTCGTGCGGTAGACTAAGTGATAT
AGCGTATTTTCAGTTTCTTCGTACCActctgcatattttttttttcttactgtaATCTTCTTCGTTTTGATATGAGCCGttc
aaaaatagatttaattctCTTCTGAGGTAAAACAAATTGTCCAATATTAGCAAGGACACATTAAAGTCTATC
TTGCGAAAATATACCTCGTACATTACGTTAAAATTCGCTTTAAAATATATGCTGCTCTGGTCTTCA
AAATTCTGTTTCATTTTAAGTTCATCGTTTTTACAATCGTGAGGGAGCCTAATATCCTTCCTATTAT
ATTGATTATAAATATCCAGCACACCCGCTAGTGTAGTTGCAAGAGTGATTTCTTTAGGGGTGTTGT
CTTTAGGAGGGTTTTCCCTAGATTCCATGGAAAGCTTTTTGTAAAGTTCTATTTGAAATGTATTAA
ACTCTGTGAGGCAATGATAGTTAATCCACATCTGTtggttgtgtttattttttttcagcagTTTGTTTAGCGTCTTTA
AGCAACTTTTCGAGGGCTTCCAATTTAATAATGTCGTATCCTATGCAAAATAAGTTGCCGCACCA
GTGGTCGTACAACATTTCGACCATACAGAAAGCATCGCGGCGTAGCGCAAGTTCGATCGCTTTCT
TTCTCTGGTTGTGCACTTCTATTTCGTTGACGCTTGAGTTCACCAGGTAATCTAAGAAATGGGTCT
CCTTCAATAAGTAGTTCAGGATATGATTCTGTTTTGCCTTGCAAGCTTTGATGATCACATCATGCT
CCGTTATACATTCGTTTCCGAGGGCATACAGATCTGCTAGCAACTGCTTATTTCGCAGCAGATCAT
GTTCGAACCATTGAACTACATTTTTAACGTCTTCTATGAATCCTTTACCGACGGCATTaagtaatatttgta
attttctttttaacgcTTTTTTTGGATCTTGTTCTTCGGACTTGGCAAGTGGCATTTTTAGGAGTgcaaattaagtaag
tatataattaataaataaattaacacacaGACAAGGGTAATAATGATGTATCGCTTCAGTCAGCCAATGGAATAA
AATGAACGTTAGGGCTTATCAATATAGTGCGCTAAGTGTGCACTTCAAAAACATTTGACCTTGTA
TAtataggtttatttataaagatgaCTA
